# High-resolution structural variants catalogue in a large-scale whole genome sequenced bovine family cohort data

**DOI:** 10.1186/s12864-023-09259-8

**Published:** 2023-05-01

**Authors:** Young-Lim Lee, Mirte Bosse, Haruko Takeda, Gabriel Costa Monteiro Moreira, Latifa Karim, Tom Druet, Claire Oget-Ebrad, Wouter Coppieters, Roel F. Veerkamp, Martien A. M. Groenen, Michel Georges, Aniek C. Bouwman, Carole Charlier

**Affiliations:** 1 Animal Breeding and Genomics, Wageningen University & Research, Wageningen, the Netherlands; 2 Unit of Animal Genomics, Faculty of Veterinary Medicine, GIGA-R &, University of Liège, Liège, Belgium; 3 GIGA Institute, GIGA Genomics Platform, University of Liège, Liège, Belgium

**Keywords:** Structural variants, Copy number variants, Whole genome sequencing, Cattle, eQTL, Linkage disequilibrium

## Abstract

**Background:**

Structural variants (SVs) are chromosomal segments that differ between genomes, such as deletions, duplications, insertions, inversions and translocations. The genomics revolution enabled the discovery of sub-microscopic SVs via array and whole-genome sequencing (WGS) data, paving the way to unravel the functional impact of SVs. Recent human expression QTL mapping studies demonstrated that SVs play a disproportionally large role in altering gene expression, underlining the importance of including SVs in genetic analyses. Therefore, this study aimed to generate and explore a high-quality bovine SV catalogue exploiting a unique cattle family cohort data (total 266 samples, forming 127 trios).

**Results:**

We curated 13,731 SVs segregating in the population, consisting of 12,201 deletions, 1,509 duplications, and 21 multi-allelic CNVs (> 50-bp). Of these, we validated a subset of copy number variants (CNVs) utilising a direct genotyping approach in an independent cohort, indicating that at least 62% of the CNVs are true variants, segregating in the population. Among gene-disrupting SVs, we prioritised two likely high impact duplications, encompassing *ORM1* and *POPDC3* genes, respectively. Liver expression QTL mapping results revealed that these duplications are likely causing altered gene expression, confirming the functional importance of SVs. Although most of the accurately genotyped CNVs are tagged by single nucleotide polymorphisms (SNPs) ascertained in WGS data, most CNVs were not captured by individual SNPs obtained from a 50K genotyping array.

**Conclusion:**

We generated a high-quality SV catalogue exploiting unique whole genome sequenced bovine family cohort data. Two high impact duplications upregulating the *ORM1* and *POPDC3* are putative candidates for postpartum feed intake and hoof health traits, thus warranting further investigation. Generally, CNVs were in low LD with SNPs on the 50K array. Hence, it remains crucial to incorporate CNVs via means other than tagging SNPs, such as investigation of tagging haplotypes, direct imputation of CNVs, or direct genotyping as done in the current study. The SV catalogue and the custom genotyping array generated in the current study will serve as valuable resources accelerating utilisation of full spectrum of genetic variants in bovine genomes.

**Supplementary Information:**

The online version contains supplementary material available at 10.1186/s12864-023-09259-8.

## Background

Structural variants (SVs) are genomic segments (> 50-bp) for which the structure between genomes differs, and may include deletions, duplications, insertions, inversions, and translocations [1]. SVs together affect more base pairs than small genetic variants (single nucleotide polymorphisms (SNPs) and small insertions and deletions (indels)), thereby have been assumed to have large phenotypic impact [2–4]. Following this idea, expression QTL (eQTL) studies in humans showed that SVs have a disproportionately high contribution to altering gene expression compared to SNPs and indels [5–7] and many functional SVs associated with various traits have been identified in humans [8]. Likewise, identifying functional SVs associated with economically important traits has been a prime interest for animal breeders. Until now, catalogue of functional SVs reported in farm animals contain many deletions that often are associated with disease traits. In contrast, duplications often are associated with distinguishable coat colours and morphologies (e.g. breed defining traits), with few exceptions [9, 10].

Discovery and genotyping of genetic variants provide a foundation for genetic analyses. In recent decades, the genomics revolution enabled accurate detection of millions of SNPs through whole-genome sequencing (WGS) technologies, and high throughput genotyping in a large number of individuals using SNP arrays. However, unlike SNPs, detection and genotyping methodologies for structural variants (SVs) have been lagging behind [11]. Array data is widely used for SNP genotyping in animal breeding, and also has the potential to detect unbalanced SVs, such as copy number variants (CNVs, a subset of SVs including deletions and duplications). Still, low resolutions and undefined breakpoints are considered major drawbacks of array-based methodologies to detect SVs [3]. Alternatively, short-read WGS data can be used to detect SVs, including CNVs and balanced SVs (e.g. inversions) at a finer resolution [1]. Despite such advancement, WGS data with low sequencing depth (e.g. <10X) suffers from low detection sensitivity, unresolved breakpoints, and low genotyping accuracy [1, 3, 11]. These issues can be alleviated by (i) exploiting WGS data with higher sequencing depth (e.g. >30X), (ii) including family samples, and (iii) confirming the discovery results using orthogonal validation (e.g. long-read sequencing data) [11]. Furthermore, the choice of SV detection methods can affect the discovery results. Some SV detection tools scan WGS data for split reads (SR) and/or discordant read pairs (DP) clusters. In contrast, other detection tools measure read-depth changes relative to the depth of genome-wide diploid regions to determine the copy number variable regions. Recent benchmark studies showed that combining these principles outperforms detection methods solely relying on a single principle (e.g. generating less false calls) [12].

A high-quality catalogue of SVs with improved detection sensitivity, including a broad size range, base-pair resolved breakpoints, and accurate genotyping can benefit genetic studies and accelerate the discovery of functional SVs. Yet, until now, lack of suitable data sets hindered obtaining a high-quality SV catalogue in the Holstein Friesian (HF), a major dairy cattle breed [13]. Absence of a high-quality SV catalogue has left some questions unanswered. Firstly, the potential for SVs for animal breeding is unknown, because it remains to be investigated whether a widely used 50K SNP genotyping array captures genome-wide SVs. Secondly, current SV catalogues based on genotyping arrays consist of large, breakpoint unresolved CNVs [14], and hence, hinder assessments of functional and phenotypic impact of SVs.

This study aimed to generate and explore a high-quality SV catalogue using WGS data obtained from a cattle family cohort (including 127 trios). We detected three different classes of SVs (deletions, duplications, multi-allelic copy number variants (mCNVs)) based on a methodology exploiting signals from both SR and DP evidence, with post hoc filtering based on the read-depth changes. Furthermore, a subset of SVs (210 deletions and 22 duplications) was validated in an independent cohort of animals using a direct genotyping approach. Using a high-quality call set, we explored population genetics features of SVs and finally, we performed in-depth characterisation of putative high impact SVs.

## Results

### Initial discovery of structural variants

We discovered SVs using short-read WGS data from 266 HF dairy cattle samples (mean coverage of 26X, min = 15X, max = 47X), using the bovine reference genome ARS-UCD1.2. The pipeline used (Smoove; https://github.com/brentp/smoove) [15] detects SVs in the individual samples based on SR and DP evidence. The number of discovered SVs per sample increased as the sequencing depth increases, suggesting the absence of spurious technical bias and high quality underlying WGS data (Figure S1). Aggregating the SVs discovered across all samples, we obtained 38,094 non-redundant SVs (17,826 deletions, 4,652 duplications, 1,811 inversions, 13,805 breakends (non-canonical type of SVs)), for which the entire cohort was genotyped. Further analyses were focused on 22,478 CNVs (17,826 deletions and 4,652 duplications), and inversions and breakends were not considered.

### Establishing a clean call set exploiting the post hoc filtering based on read-depth changes

**Fig. 1:**
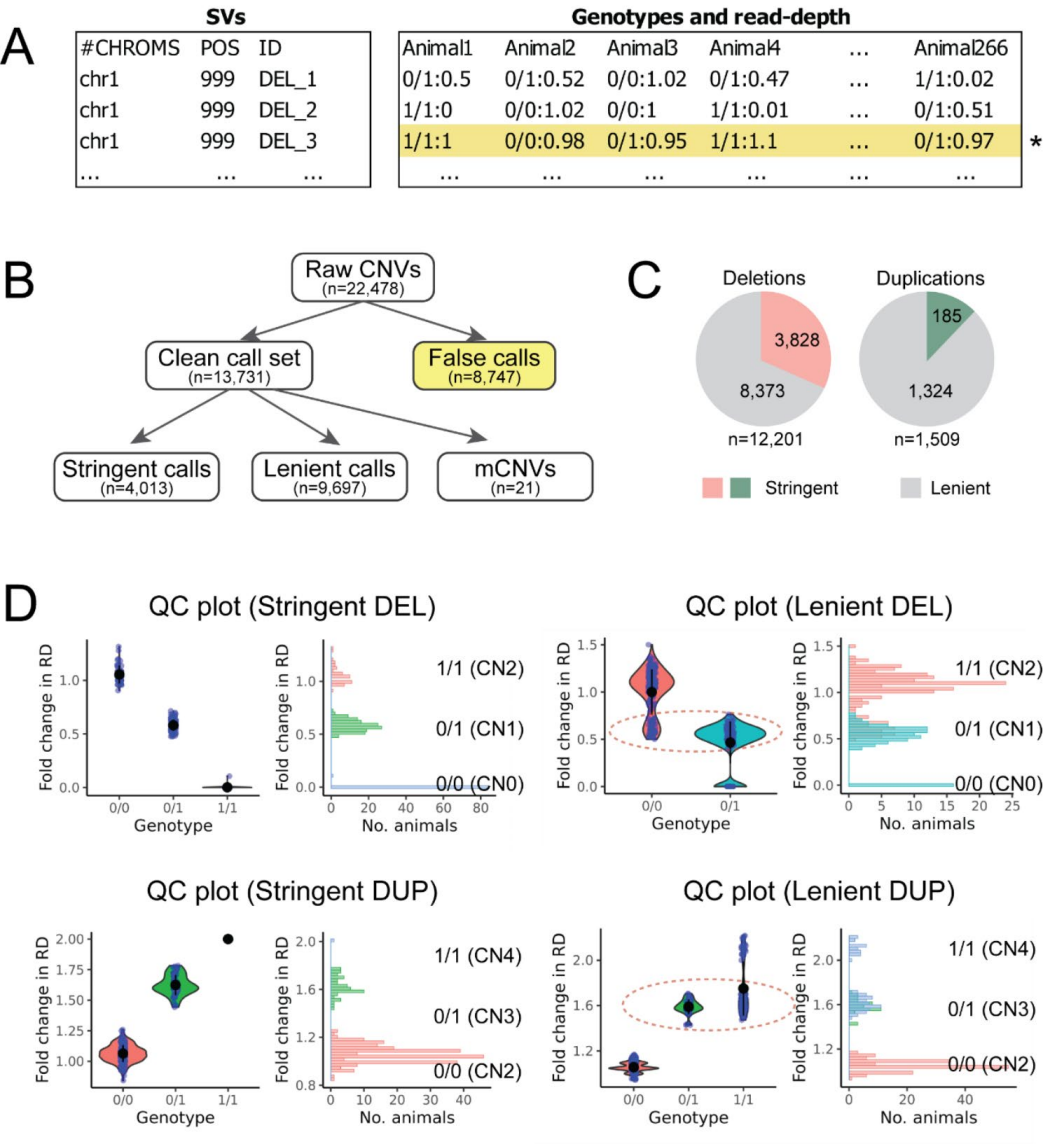
Discovery and quality control on SVs in the bovine genomes **(A)** An example of population-side SV detection results. Animals are genotyped for each site, and for CNVs, the fold-coverage change in read-depth is annotated. Marked with yellow is a spurious call where read-depth do not change according to genotypes. **(B)** An overview on filtering steps and number of calls in different call sets. **(C)** The overall CNV calls were divided into stringent and lenient call sets, exploiting the post hoc filter based on read-depth. The former is considered to be the set of accurately genotyped biallelic sites. **(D)** Quality control (QC) plots were generated for all CNVs exploiting the genotype and read-depth information. The panel on the left side shows an example of a stringent site where animals’ genotypes and read-depth are unambiguously assigned. Each blue dot represents a sample. The black dots and vertical bars in the violin plot represent the mean and one standard deviation. The right panel represents the read-depth distribution for each GT group. The QC plot for a lenient site is shown on the right side. In such a case, read-depth distribution of animals genotyped as 0/0 and 0/1 are overlapping (marked with a red dotted circle), indicating inaccurate genotyping results

To obtain a high-quality SV call set, we filtered out spurious SVs from the initial call set which contained 17,826 deletions and 4,652 duplications. After removing (i) SVs smaller than 50-bp, (ii) non-polymorphic SVs, and (iii) putative assembly errors, we retained 21,737 SVs (17,096 deletions and 4,641 duplications; see materials and methods). Afterwards, we refined our call set by utilising post hoc read-depth based filtering. CNVs can be genotyped by SR and DP evidence as done by our current pipeline [15], but also can be inferred from read-depth changes [16]. Hereafter the genotyping done based on SR and DP evidence is referred to as reads-based genotyping, as opposed to read-depth based genotyping. In our pipeline, the read-depth fold-coverage of each CNV was annotated [16]. For example, a heterozygous deletion with read-depth of 15X, relative to the 30X coverage in non-SV region was assigned the fold-coverage of 0.5. In contrast, a duplication where the affected region amounts to the mean coverage of 45X, opposed to the 30X coverage in non-SV region was assigned the fold coverage of 1.5. To utilize the read-depth annotation for the site-level filtering, we (i) calculated mean read-depth fold-coverage depending on genotypes and (ii) generated a summary QC plot for all CNVs (see Fig. 1D for examples). By manually inspecting all CNVs on chromosome 1, we set rules to filter out calls with spurious read-depth fold-coverage changes. For instance, for each deletion, mean read-depth fold-coverage in heterozygous deletion carrier is expected to be smaller than that of wild type animals. If this relation was reverse, we filtered them out (see materials and methods for detailed explanation on filters). After excluding CNVs with spurious read-depth fold-coverage, 12,201 deletions and 1,530 duplications were retained. While manually inspecting QC plots of all CNVs, 21 duplications were re-classified as mCNVs based on their multi-modal read-depth distributions. These loci had more than three read-depth peaks, which implied more than two alleles segregating (Figure S2). Hereafter, a total of 13,731 CNVs (12,201 deletions, 1,509 duplications, and 21 mCNVs) that passed the preliminary read-depth filters is referred to as a “clean call set” (Fig. 1B). Together, these CNVs were in a size range between 50-bp and 424-kb (Figure S3). Overall, a median number of 5,252 CNVs (4,865 deletions and 387 duplications) was discovered per genome.

### Differentiating the clean call set into stringent and lenient calls

**Fig. 2:**
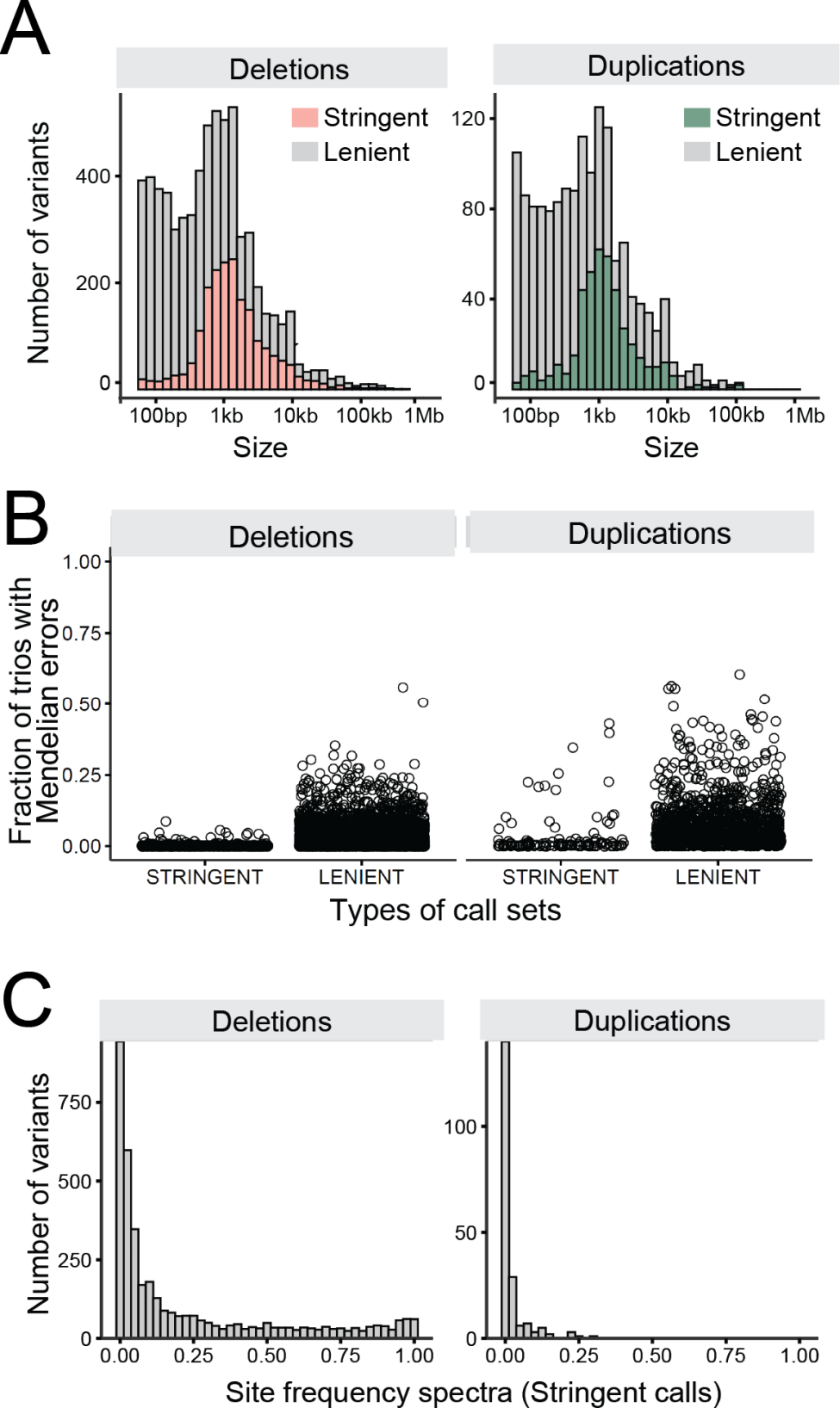
Summary of CNV call sets and quality indicator metrics **(A)** Length distribution of the stringent and lenient call sets. **(B)** Mendelian error fractions obtained for each CNV site are shown for stringent and lenient call sets. **(C)** Site frequency spectra of stringent CNVs

The 13,731 CNVs belonging to the clean call set were further scrutinized (Fig. 1). If all samples are accurately genotyped and the read-depth fold-coverage reflect the true underlying genotypes, we expect to see a mixture of two or three non-overlapping read-depth distributions matching genotypes. Alternatively, a variable distribution of read-depth within a genotype might indicate inaccurate genotyping (Fig. 1D). Hence, we divided the clean CNV call set into a “stringent” call set, with CNVs of which their read-depth fold-coverage corresponds unambiguously to the reads-based genotypes, and a “lenient” call set with CNVs of which their read-depth fold-coverage does not always match with the reads-based genotypes. The stringent call set consisted of 3,828 deletions and 185 duplications which contained accurately genotyped biallelic CNVs, mostly larger than 500-bp (Fig. 2A). On the contrary, CNVs in the lenient call set consisted of 8,373 deletions and 1,324 duplications and were often (i) small (< 500-bp), relying only on evidence from soft-clipped reads, hence did not manifest clear read-depth fold-coverage changes depending on genotype, (ii) incorrectly genotyped due to a complex local genomic context (e.g., discordant read pairs in repeat-rich regions lead to low mapping quality, thus were not taken into account in genotyping). Additionally, the genomic neighbourhoods (10-Kb flanking regions) of the stringent and lenient call sets showed different features. A fewer number of genes and repeats were present in the flanking regions of the stringent CNVs (0.7 gene and 44.7 repeats per stringent CNV), compared to lenient CNVs (1.1 genes and 64 repeats per lenient CNV).

Moreover, some duplications showed evidence of multiplication events. Multiplication loci harbour structural alleles containing copies numbers (CN) higher than two and are not necessarily biallelic, meaning that more than two structural alleles are segregating for a given locus. A recent study reported such multi-allelic CNV (likely resulting from multiplication events) associated with clinical mastitis in HF population [17], which harbours alleles with CN 1, 4, 5 and 6. Animals heterozygous for this locus (e.g. CN1/CN4) were genotyped as homozygous alternative for a duplication by SVtyper which assumes biallelic loci by default (Figure S4). After the filtering steps, the numbers of SVs in the clean call set, the stringent call set, and the lenient call set were no longer strongly determined by the sequencing coverage of the samples. This finding suggests that the large number of variants discovered in the high sequencing depth samples may be due to many false calls, which are now excluded after rigorous site-level quality control (QC) (Figure S5).

Subsequently, the quality of each CNV call set was evaluated using the family structure present in the data set (127 trios). A quality metric was coined expressing a fraction of trios having Mendelian errors at each site (e.g., with 15 out of 127 trios manifesting Mendelian errors, the fraction corresponds to 0.12). As expected, the stringent call set showed lower Mendelian errors overall than the lenient call set (Fig. 2B). Notably, duplications showed higher error rates than deletions in both call sets, suggesting that duplications are prone to having more genotyping errors even when strict filters are applied. One caveat of the Mendelian error fraction quality metric is that it might be confounded with the allele frequency. That is, a rare SV may seem to have low Mendelian errors, due to low number of occurrences of the SV itself. However, Mendelian error fraction did not increase along with the allele frequency for both stringent and lenient deletions, confirming that the current quality metric is not affected by the allele frequency (Figure S6). We inspected the site frequency spectra limiting to the stringent call set, which was considered to contain accurately genotyped CNVs that were relatively skewed towards large events (Fig. 2A). Both deletions and duplications showed similar allele frequency spectra in a sense that they showed many rare variants. Notably, the majority of the stringent duplications were rare, where a handful of them reached allele frequency of ~ 0.25 (Fig. 2C). Finally, we inspected the breakpoints of CNVs. In total, 68% of CNVs had single base resolved breakpoints, both in the stringent and the lenient call set. The high number of single base resolved breakpoints in the lenient call set gives confidence that the CNV are correctly called, despite the low genotyping accuracy.

### Validating the SV discovery results using direct genotyping approach

**Fig. 3:**
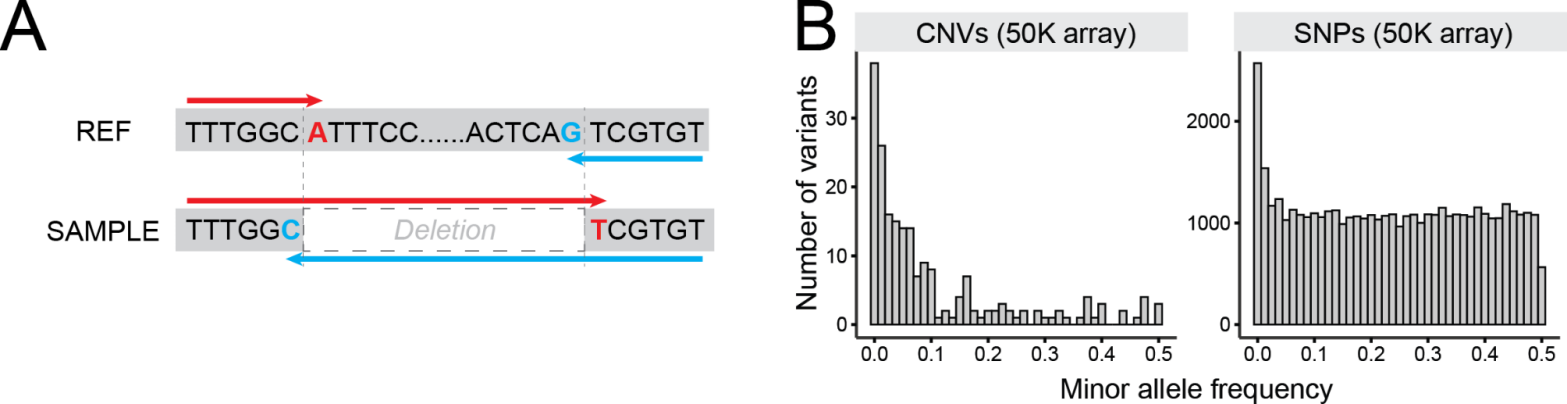
Direct genotyping approach and results **(A)** A schematic overview on primer design. To genotype a deletion, a forward assay can target A (marked with red) in the reference, whereas T will be targeted in deletion carriers. A reverse assay can target G (marked with blue), whereas C will be targeted in deletion carriers. **(B)** Site frequency spectra of CNVs and SNPs obtained from the validation data (50 K SNP genotyping array)

SVs discovered in the WGS data set, if validated in animals in the same population other than the animals of the discovery cohort, would confirm that the variant of interest is segregating in the population. To this end, we aimed at validating a subset of the WGS CNVs by directly genotyping the breakpoints of CNVs in animals not overlapping with the WGS cohort. Among the CNVs in the catalogue, breakpoints of 9,642 CNVs had a single-base resolution; thus, genotyping probes could be designed (Fig. 3A, see methods). Of these, we designed probes for 371 CNVs (342 deletions and 29 duplications) which appeared in non-repetitive regions and added them to the custom part of the EuroGenomics custom genotyping array [18], which include ~ 50K SNPs (hereafter referred to as 50K SNP array for brevity). Genotyping was done in 815 HF animals, not overlapping with the WGS animals. Of the 284 CNVs (262 deletions and 22 duplications) that passed the QC criteria (call rate per sample > 0.99 and call rate per variant > 0.99), 211 deletions and 19 duplications were segregating in the genotyped population (allele count ≥ 1). The remaining 54 probes did not segregate in the population (allele count = 0), despite passing the QC criteria, indicating that either (i) the targeted CNVs were not present among the 815 HF animals, or (ii) the CNV-targeting probes did not work. The allele frequency of CNVs was skewed towards rare alleles, compared to that of the 50K SNPs obtained from the same array (Fig. 3B), yet were similar to what is observed in the discovery data set (Figure S7). In short, the CNV genotyping results independently validated at least 62% of the CNVs selected from the WGS CNV catalogue (229/371). The 229 validated CNVs confirm that these are variants segregating in the population, which may be exploited in selection.

### Comparison with other call sets

We compared the current WGS-based SV catalogue with a bovine pangenome SV call set established from 57 breeds (consisting of 898 animals; 148,371 deletions and 7,468 duplications) [19]. The overlap between the two call sets was limited: 2,965 deletions (corresponds to 24.3% of deletions in the current call set; 2,965/12,201) and 707 duplications (corresponds to 46.9% of duplications in the current call set; 707/1,509) overlapped based on a minimum of 95% reciprocal overlap. Such limited overlap justifies a deep characterization of SV focusing on a single breed. Subsequently, the current call set was compared with an array-based catalogue generated from 315 animals from the same HF population [14]. The concordant CNVs between the WGS- and array- catalogues were mostly large CNVs (231 concordant CNVs, mean size of 33-Kb, min size = 1.2-kb, max. size = 402-Kb). Given that the size of WGS-based CNVs were discovered mostly around or smaller than 1-kb size (Fig. 2A), the WGS-based catalogue seems to contain a large number of finer scale variants undiscovered based on the array data, likely because of low resolution.

All things considered, the current SV call set represents major advancements. Firstly, SV detection in WGS data resulted in improved resolution thereby discovering many small SVs which was not discovered in the array-based SV catalogues. Secondly, 68% of SVs were defined with single base resolved breakpoints, which can be beneficial in investigating functional impact of SVs. Thirdly, our catalogue contains two subsets (stringent and lenient) which represent different levels of confidence in quality and genotyping accuracy. Thus, this high-quality call set stands for a powerful resource for population and functional analyses.

### CNV-SNP linkage disequilibrium in the WGS data set

**Fig. 4:**
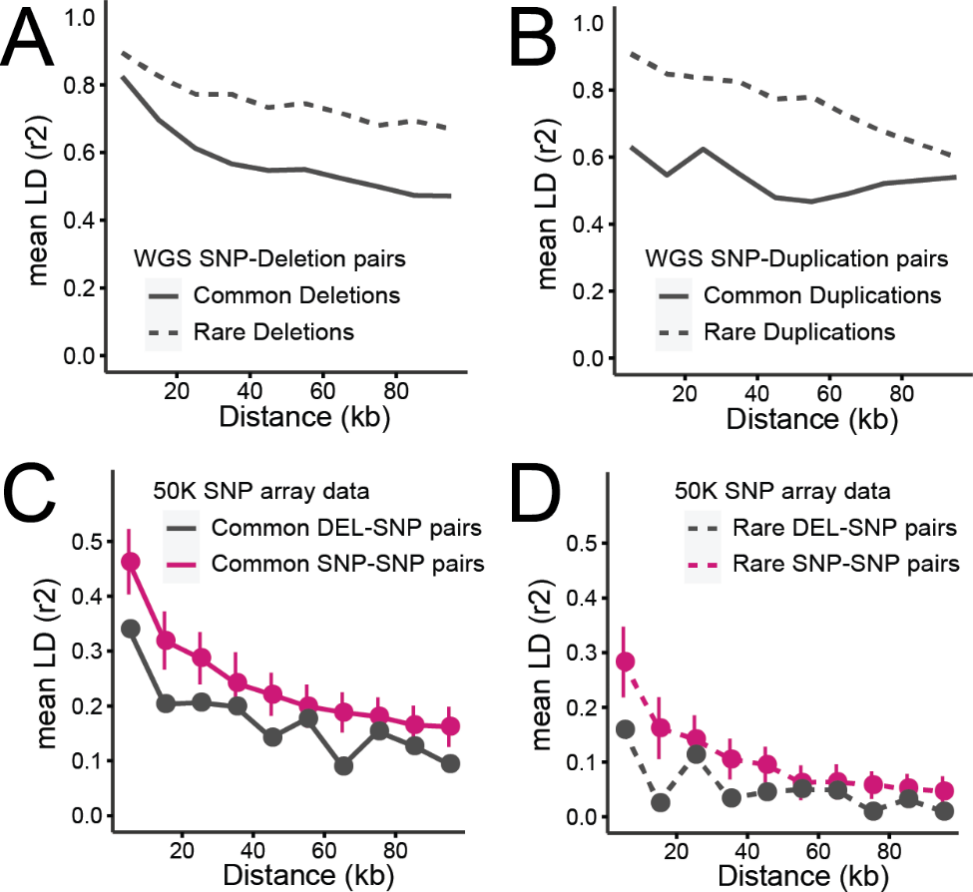
Linkage disequilibrium between SNPs and CNVs **(A)** Mean r^2^ obtained from deletion-SNP pairs discovered in WGS data is displayed as a function of inter-marker distances. SNPs paired with common deletions (MAF ≥ 0.05) are marked with a solid line, whereas SNPs paired with rare deletions (MAF < 0.05) are marked with a dotted line. **(B)** Mean r^2^ obtained from duplication-SNP pairs discovered in WGS data is shown. The legend is the same as panel (A). **(C)** Mean r^2^ obtained from 50 K SNP genotyping array is displayed for common variants only (MAF ≥ 0.05). The SNP-SNP pairs are marked with a solid magenta line, and DEL-SNP pairs are marked with a solid grey line. The SNP-SNP pairs outnumbered the DEL-SNP pairs. To keep the comparison not influenced by the difference in the number of pairs, a subset of SNP-SNP pairs, equivalent to the number of DEL-SNP pairs, was made 1,000 times, and the mean and the standard deviation are displayed in the figure. **(D)** Mean r^2^ obtained from 50 K SNP genotyping array is displayed for rare variants only (MAF < 0.05). Legends are identical to the panel (C)

Although a handful of CNVs associated with complex traits have been delineated at a molecular level [20, 21], large scale genomic analyses are often centred around utilising SNPs, leaving CNVs unexplored. In theory, if a CNV is in high linkage disequilibrium (LD; e.g. r^2^ > 0.8) with SNPs, those SNPs should capture the CNV, serving as a tagging marker. Hence, we calculated pairwise LD (r^2^) between CNVs and SNPs obtained from WGS data to evaluate whether SNPs tag CNVs. First, we focused on the stringent CNV call set, as it contains accurately genotyped biallelic CNVs. In this call set, 97% and 93% of the deletions and duplications, respectively, were captured by sequence level SNPs, and the CNV-SNP LD broke down as the inter-marker distance increases (Fig. 4A and B). Our results showed that even rare CNVs (minor allele frequency (MAF) < 0.05) were well tagged by SNPs, likely due to rare SNPs and CNVs occurring on the same haplotype that is private to particular families. Next, we investigated the LD in the lenient CNV call set, and the fraction of tagged CNVs reduced to 83% and 61% for deletions and duplications, respectively (Figure S8). Of note, the mean SNP-CNV distance in the stringent and lenient call sets were 25.9-Kb and 27.8-Kb, respectively. The discrepancy in LD between stringent and lenient call sets suggests that the lower degree of LD in the lenient call set arises from inaccurately genotyped CNVs, instead of actual lack of tagging SNPs. Additionally, we calculated LD between CNVs and SNPs called from WGS data, but when the SNP density was reduced to that of a 50 Karray (see below). The 50K SNP set captured 13.4% and 3.3% of the deletions (1,631/12,201) and duplications (50/1,509), respectively. Finally, we expected that the mCNV-SNP LD would be generally low given (i) that biallelic SNPs would be in partial LD with a multi-allelic variant and (ii) high genotyping error arising from our pipeline that will blindly assign biallelic genotypes to multi-allelic loci. Indeed, of 21 mCNVs, only 40% were in LD with SNPs (r^2^ > 0.8) based on the biallelic genotypes assigned by SVtyper. Interestingly, these 21 mCNVs included a 12-kb mCNV associated with clinical mastitis, partially overlapping with the group-specific component (*GC*) gene (hereafter referred to as *GC* CNV) [17]. A previous study of the *GC* CNV reported presence of tagging SNPs (both within and outside the CNV) when the four structural alleles segregating at this locus are grouped into two (wildtype or multiplicated alleles). However,  we did not detect tagging SNPs for the GC CNV in the current analyses, likely because the standard biallelic genotyping could not take into account the underlying structural alleles of the GC CNV, leading to false genotyping (Mendelian error fraction = 0.33).

### CNV-SNP LD in the array data set

Our results from the stringent call set showed that sequence level SNPs could capture most of the biallelic CNVs as long as the biallelic CNVs are accurately genotyped. However, in animal breeding, large-scale genomic analyses (e.g. genomic selection) rely on 50K, or lower density SNP data. To assess whether array level SNPs capture CNVs, we investigated DEL-SNP LD based on genotypes of 50K SNPs and 211 deletions directly obtained from our custom 50K SNP array, explained above. In the 50K genotyping array data set, both DEL-SNP and SNP-SNP pairs showed LD decay where the degree of LD declines as a function of inter-marker distance. Intriguingly, DEL-SNP pairs showed lower mean LD than SNP-SNP pairs, regardless of the allele frequency (Fig. 4C and D). Finally, we checked the fraction of variants that has tagging SNPs (r^2^ > 0.8): 14.1% of studied SNPs (6,068/42,973) and 19.4% of the deletions (41/211) had tagging SNPs. None of the 19 duplications had tagging SNPs.

### Predicted functional impact of SVs

**Fig. 5:**
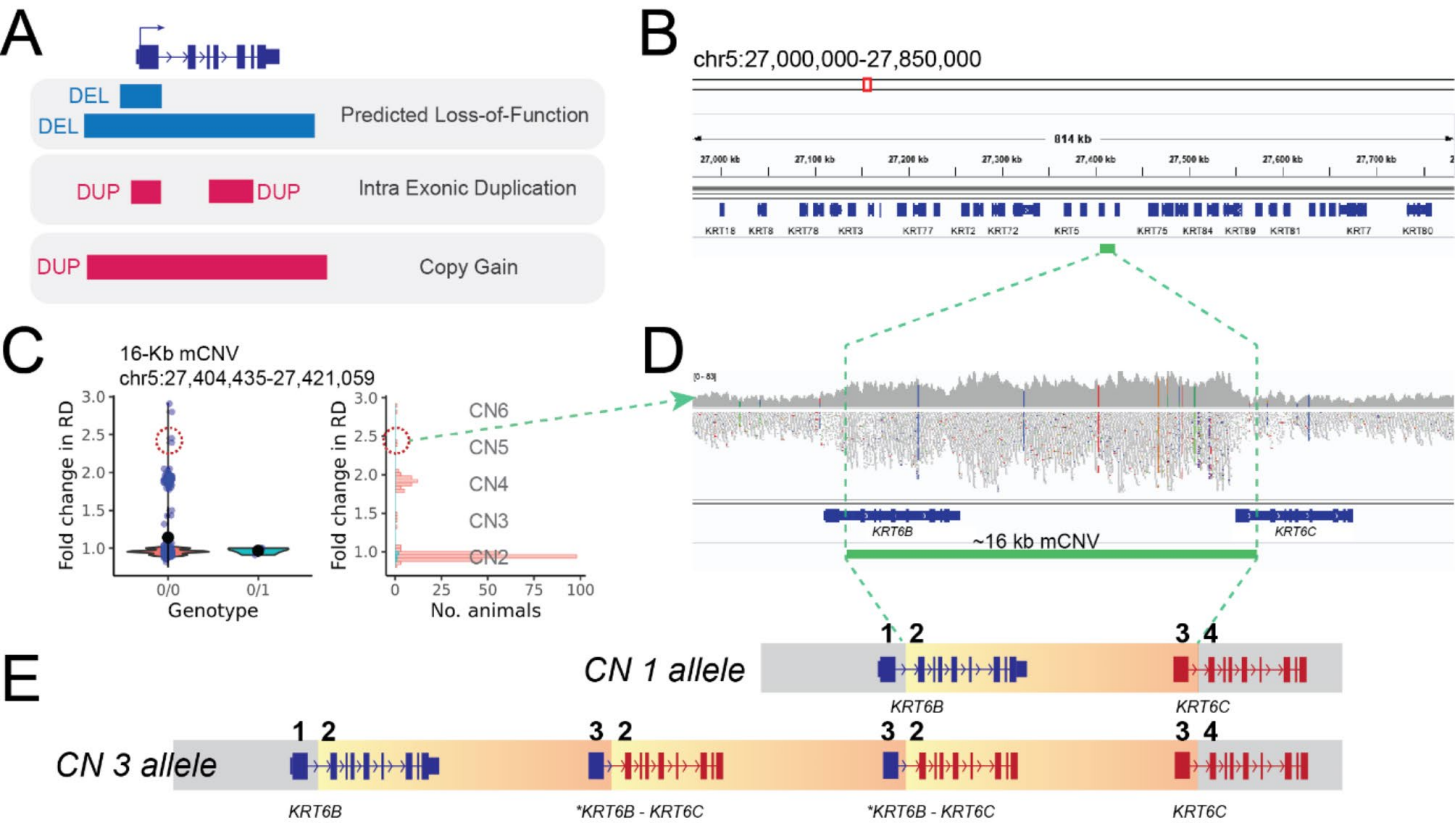
CDS disrupting SVs **(A)** Three different categories of CDS disrupting SVs. CDS disrupting deletions and insertions can lead to loss-of-function variants. If affecting an entire gene, duplications are equivalent to obtaining an extra copy of a gene (copy gain). However, partial duplication of a gene may have different consequences depending on the context. Figure adapted from [22]. **(B)** A 16-kb mCNV was found in the Keratin gene-rich region, harbouring more than 20 keratin genes, in the chr5:27 Mb region (marked with green). This mCNV affects two keratin genes, *KRT6B* and *KRT6C*. **(C)** The QC plot of the 16-Kb implied that diploid CNs range between 2 and 6, yet reads-based genotype indicated inaccurate genotyping. **(D)** WGS data of one of the mCNV carriers was inspected (diploid CN 5). Increased sequencing coverage supports the presence of multiple copies of the 16-kb segment. **(E)** The Tandem arrangement of the 16-kb segment can give rise to a novel fusion gene made of part of *KRT6B* and *KRT6C* (marked with an asterisk; shown in blue and red). In this panel, we depicted a putative tandem arrangement of the haploid CN3 allele

The functional consequence of SVs varies depending on many factors, including SV types, event sizes, the overlap with coding sequences (CDS). In the case of deletions, they may overlap or occur within CDS of a gene, thus leading to loss-of-function of the gene. In contrast, duplications may have different consequences depending on overlap with CDS. For instance, a duplication partially overlapping with a coding gene (e.g., overlapping with a subset of exons), may alter transcript(s), whereas a duplication encompassing an entire gene may end up increasing gene expression in theory. Therefore, following previous literature [21], we categorised CDS overlapping SVs into three classes: (i) predicted loss-of-function (pLoF) for CDS disrupting deletions, (ii) intragenic exonic duplication for duplications with partial genic overlap, and (iii) copy gain for duplication encompassing entire gene(s) (Fig. 5A).

Our SV catalogue overlapped with the CDS of 426 genes (342 pLoFs, 41 copy gains, 50 intragenic exonic duplications; some genes were affected by more than one SVs), and each individual had on average 88 predicted loss-of-function, 7.8 copy gain, and 8.1 intragenic exonic duplication events. The list of CDS disrupting SVs contained three high impact SVs: (i) a predicted loss-of-function event by a 3.3-Kb deletion ablating *FANCI* gene, causing foetal death and brachyspina [23], (ii) a predicted loss-of-function event by a 138-Kb deletion ablating *TFB1M* gene, which was associated with a lethal haplotype mapped in HF population [24], and (iii) an intragenic exonic duplication event by a 12-Kb mCNV overlapping with the last exon of *GC* gene, associated with mastitis resistance [17]. As expected, common CDS disrupting SVs (MAF > 0.05) were often affecting genes belonging to large gene families (e.g., olfactory receptors), whereas rare SVs often disrupted essential genes without paralogues. For example, we discovered a singleton 50-kb deletion ablating *Centromere Protein C* gene (*CENPC*), which was shown to be recessive lethal in a mouse knock-out study [25]. Moreover, we identified a 16-kb intragenic exonic duplication event in the BTA5 27.4 Mb region that harbours a large repertoire of keratin genes (Fig. 5B). This intragenic exonic duplication event was classified as mCNV based on the multi-modal read-depth distribution, which indicated diploid CNs between 2 and 6 (Fig. 5C). Furthermore, the QC plot implied inaccurate genotyping (e.g., mCNV carriers with high read-depth were genotyped as 0/0). Close inspection of carrier animals supported the presence of the mCNV (elevated sequencing coverage; Fig. 5D), however the reads spanning over breakpoints had low mapping quality leading to inaccurate genotyping (Figure S9). This 16-kb mCNV disrupts two keratin genes that are in the same orientation (*KRT6B* and *KRT6C*), and thus can give rise to a novel fusion gene (Fig. 5E). In such case, a diploid CN6 animal is expected to have intact *KRT6B* and *KRT6C* genes and 4 copies of *KRT6B-KRT6C* fusion genes.

### Molecular characterisation of *SV-eQTL*

A recent human SV catalogue showed that most SVs are under purifying selection, thus segregating at low allele frequency, except duplications encompassing entire gene(s) [22]. Therefore, we focused on the 41 copy gain events aiming at identifying functional duplications. The underlying assumption of functional copy gain events is that an extra copy of a gene can increase gene expression. Thus, mapping SV expression QTL (SV-eQTL) for copy gain events seemed a plausible approach to elucidate molecular contribution of SVs. Before SV-eQTL mapping, we prioritised copy gain events harbouring genes that previous studies reported associations with economically important traits in cattle. Based on the literature, we found two promising duplications. The first is an 85-kb duplication harbouring *Orosomucoid 1* gene (*ORM1*; chr8:103,486,032–103,571,582) (Figure S10A-D). *ORM1* is predominantly expressed in liver and encodes acute-phase plasma protein, and has been shown to be upregulated in response to acute inflammation [26]. In dairy cattle, an increased *ORM1* expression in postpartum cows was associated with decreased feed intake [27, 28]. The second is a 150-kb duplication harbouring *Popeye Domain Containing 3* gene (*POPDC3*; chr9:44,725,475 − 44,875,600; Figure S10E-H). *POPDC3* is involved in skeletal muscle tissue development and is broadly expressed in multiple tissues [29]. This 150-kb duplication was associated with hoof health traits in Canadian HF population; however the effect direction was not reported [30].

**Fig. 6:**
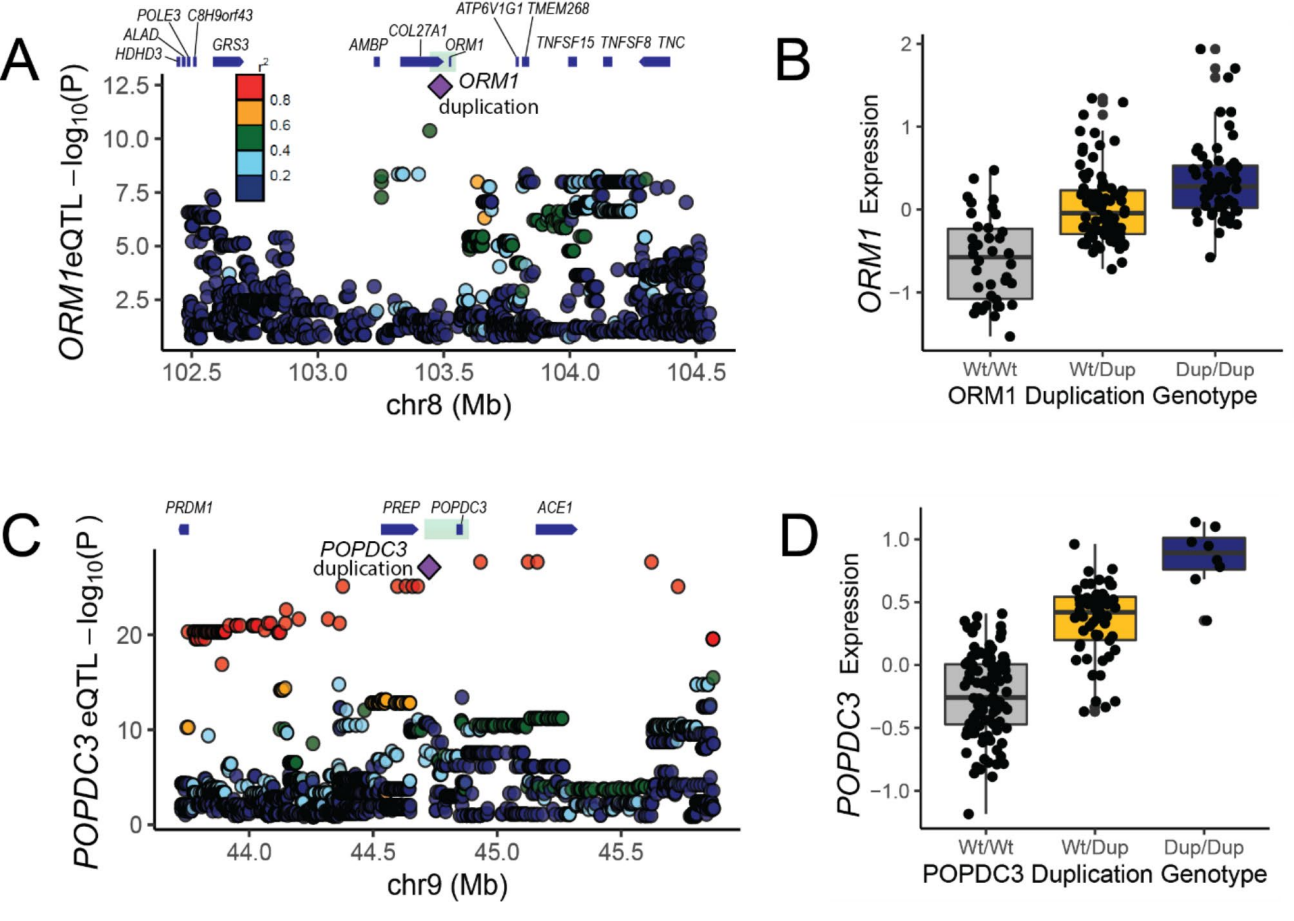
SV eQTL mapping results **(A)** eQTL mapping result for *ORM1.* The *ORM1* duplication is marked with purple diamond. The colour scale indicates the degree of pairwise LD (r^2^) between the *ORM1* duplication and other SNPs. Green translucent box marks the duplication. **(B)** The box plot shows altered *ORM1* expression depending on the *ORM1* duplication genotypes. **(C)** eQTL mapping result for *ORM1*. Legend is same as panel (A). **(D)** The box plot shows altered *POPDC3* expression depending on the *POPDC3* duplication genotypes

We proceeded with SV-eQTL mapping exploiting BovineHD genotype and liver RNA-seq data obtained from postpartum day 14 dairy cows (n = 175). To associate the gene expression with the SVs detected from the WGS data, we generated an imputation panel consisting of SNPs and SVs discovered in 266 WGS animals (see method; Figure S10I). The BovineHD genotype was imputed to sequence level SNPs and SVs, and then the imputed genotypes were associated with gene expression. The *ORM1* duplication was well imputed and ranked as the top variant for *ORM1* eQTL (Fig. 6A and B). The same procedure was applied to *POPDC3* duplication and likewise, the imputed *POPDC3* duplication was among the top eQTL variants for *POPDC3* (Fig. 6C and D and Figure S10J). Furthermore, bovine liver ChIP-seq data (H3K27ac and H3K4me3 [31]) confirmed the presence of promoters for these genes, providing a mechanistic explanation on these liver SV-eQTL (Figure S10 C, G). Thus, it is plausible that these duplications lead to a true copy gain event of the cognate genes, ultimately leading to an increased gene expression (Fig. 6). Extrapolating the literature, we could hypothesise that the *ORM1* duplication allele, which leads to high *ORM1* expression, will decrease the feed intake in postpartum cows [27, 28]. In mice, administration of exogenous ORM supressed food intake, via binding leptin receptors, which induce activation of signal transducer and activator of transcription 3 (STAT3) signalling [26]. A recent dairy cattle study showed that high ORM expression suppressed postpartum feed intake, yet without triggering STAT3 signalling, leaving the underlying appetite suppression mechanism elusive [28]. It is worth noting that this variant is highly frequent (MAF = 0.49), despite its association with the reduced feed intake, which is considered detrimental for postpartum cows. One possible explanation could be that this variant is under balancing selection. In an attempt to identify target trait(s) under selection, the animal QTL database was screened [32], however, there was no QTL reported in the region of interest.

## Discussion

In this study, we used 266 sequenced Dutch dairy cattle genomes to discover SVs. SV discovery and our understanding of SVs have been hindered by low detection sensitivity and inaccurate genotyping issues often arising in low sequencing depth samples [11]. In our study we focused on deletions and duplications, as these can be called most accurately with the type of sequence data we had (Illumina short read sequencing with a fragment length of 550-bp and a read length of 100-bp). Insertions were ignored because comparison of insertion callers showed that at least 60X coverage was needed to reach the maximal sensitivity for insertion detection [12]. Structural variant detection tools relying solely on a single detection principle were shown to generate many false positive calls compared to ensemble callers [12, 33], resulting in a low-quality call set. To address these issues, we discovered SVs in a large-scale healthy bovine family cohort WGS data. It is worth noting the unique pedigree structure in the data set (127 trios) provided an independent measure of quality evaluation (i.e. Mendelian errors), but also the sequencing was done at a relatively high depth (mean sequencing depth = 26X), compared to other studies that investigated SVs in livestock species [34–37]. Detection of SVs can benefit from high coverage sequencing data in two ways. First and foremost, it will inevitably improve detection sensitivity. There are more split reads and discordant read pairs evidence supporting SVs for a given locus, leading to an increased number of discovered variants. Secondly, the read-depth can be measured accurately, hence can be exploited to filter out spurious false positive calls (e.g., heterozygous deletion without reduction of read-depth can be filtered out). Of these, we exploited read-depth fold-coverage annotation to distinguish the clean CNV calls into stringent calls with high genotyping accuracy. Thus, if future studies aim to investigate SVs using allele frequency-based analytical tools (e.g., Fst), the stringent call set may be a good starting point as it contains accurately genotyped CNVs. It is worth noting that duplications had overall higher Mendelian errors than deletions. If a deletion is present in one’s genome, it can either be a heterozygous deletion (diploid CN1) or homozygous deletion (diploid CN0). Contrary to this, duplications can be biallelic or multi-allelic. Also, multiplication events are often detected as duplications. In these cases, diploid CNs may not ascend sequentially, as explained earlier (Figure S4). For a multiplication locus harbouring structural alleles with CNs 1 and 4, heterozygous animals (e.g., CN1/CN4) are genotyped as homozygous for duplication, due to overwhelmingly large number of discordant reads – resulting in a Mendelian error. Thus, exploiting duplications may require more effort to characterise the true underlying CN states, requiring high sequencing depth to identify read-depth differences. High number of false positives and negatives in SV discovery makes it crucial to perform post-discovery evaluation [11]. Commonly used orthogonal validation methods include long-read WGS and PCR amplicons [22, 38–40]. However, often these validations can be costly and time-consuming, and above all, the availability of DNA material can be a bottleneck. Instead, to some extent, we bypassed these issues by incorporating CNV targeting probes into the 50K SNP array that is routinely used in livestock breeding programmes. This approach allowed us to obtain accurate genotypes of CNVs and SNPs simultaneously. Future use of the 50K array presented in this study, for a massive genotyping of ~ thousands of animals will lead to opportunities (i) to evaluate a functional impact of individual CNV or (ii) to evaluate an overall genetic contribution of CNVs, relative to 50K SNPs. For example, our results showed that ~ 62% validated CNVs confirmed that these are population variants, suitable to be used for selection. Yet, the remaining ~ 38% of CNVs were not validated in the current cohort of 815 HF animals. Without precluding genetic drift or technical issues (e.g., suboptimal design of probes) as potential causes of the 38% of the CNVs that were not detected in the validation cohort, we consider rare CNVs private to some families in the discovery cohort might also explain the non-validated CNVs. If the latter counts for the majority of the non-validated CNVs in the current data, it is likely that the 62% validation rate is a lower-bound, and a larger validation cohort may increase the validation rate.

Our and others’ work demonstrated a large repertoire of functional SVs, many of which are of interest for livestock breeding [9, 10]. In livestock breeding, the genetic merit of animals is estimated based on the genomic prediction that exploits 50K SNPs. Thus, whether the 50K SNPs fully capture the variation from CNVs is a prime question. The CNV-SNP LD shown in our WGS data set revealed that most CNVs in the stringent call set have tagging SNPs (97% deletions and 93% duplications; Fig. 3 and Figure S8), higher than recent reports in human SV studies comprising of > 10,000 genomes of diverse ethnic backgrounds [22, 40]. Unlike human studies, we studied a family cohort from a single cattle breed, likely leading to the upper bound of the LD. After removing the 127 offspring from the trio data to limit the family structure, the LD between CNV and SNP in WGS data was still high (97% and 91.5% of stringent deletions and stringent duplications had tagging SNPs with r^2^ > 0.8). However, the DEL-SNP LD in the SNP array data set was lower, because (i) the genotyped animals were unrelated and (ii) the MAF of SNPs and deletions did not match well – CNVs were skewed towards rare variants, whereas SNPs were uniform across the range of MAFs, unlike the sequence level SNPs that include variants from a full spectrum of allele frequencies (Fig. 3B). Interestingly, fraction of SNPs or deletions that have at least one tagging SNPs were 14.1% and 19.4%, respectively. In contrast, 19 duplications obtained from the 50K array were all rare variants and none of them had tagging SNPs. These findings together are in agreement with a previous study that underlined that duplications are not as well captured by 50 KSNPs compared to deletions and SNPs [36]. Based on these findings, large scale genomic analyses aiming at investigating CNVs may consider the followings. The first is to exploit imputed sequence level SNPs that tag CNVs, instead of relying on 50K density SNPs obtained from SNP arrays. We expect that this approach will work well for SNPs that tag common CNVs, however, in reality, many CNVs are rare and so are their tagging SNPs, posing difficulty in imputation. Also, this approach will work well for the CNVs that are accurately genotyped in the sequenced reference population (e.g., stringent calls). The second approach is to impute the CNVs detected from WGS data. We think although this approach may work well for many deletions, particularly the stringent ones, duplications may not benefit much from it. As elaborated earlier, non-canonical duplications (multiplications and multi-allelic loci) showed high Mendelian errors, which can be extrapolated into low imputation accuracy. The third approach is to directly genotype CNVs as shown in the current study (adding CNV targeting probes in routinely used SNP arrays). This option is an economic option to obtain SNP and CNV genotypes from a large cohort. Despite this benefit, this approach is limited to CNVs located in relatively clean regions, and ones that do not involve repetitive sequences at breakpoints. It is worth noting that, the *ORM1* duplication, one of the functional SVs this study reports, did not have tagging SNPs either at 50K density or sequence level SNPs (data not shown), hence the first approach is not sufficient to capture it. Also, it was classified as a lenient duplication, implying suboptimal genotyping accuracy (33 out of 266 animals showed genotype and read-depth discrepancy), whereas the direct genotyping approach showed accurate genotyping results (data not shown). Together, the *ORM1* duplication demonstrates the complexity and challenges in incorporating SVs in routine genetic analyses. Lastly, although not covered in this study, haplotype-based approaches were shown to capture CNVs well [20, 21, 41], hence can be an alternative choice.

Due to large event sizes, a single SV may have larger effect, compared to smaller variants [22, 40]. Under such circumstances, SVs with deleterious effects, if affecting haplo-insufficient gene(s), are expected to be purged rapidly. Hence, it is assumed that most SVs would have a benign effect, unless they confer an adaptive advantage [2]. Our SV catalogue contains 426 genes affected by SVs, where each animal carries on average > 100 affected genes. Mapping deleterious variants can be done exploiting (i) a phenotype driven approach (e.g., GWAS), which requires high allele frequency, and (ii) a genotype driven approach (e.g. homozygosity depletion mapping), which requires a very large genotyped population and high allele frequency. Since the study population is a healthy family cohort of modest size (n = 266), it was unlikely to discover rare recessive lethal SVs using either of the two approaches mentioned above. This does not preclude that recessive lethal SVs are segregating in the current population. We confirmed that two known recessive lethal deletions (*FANCI* deletion [23] and *TFB1M* deletion [24]) are segregating in the current population, thus serving as positive controls; however, as expected, we did not see any homozygous carrier of these deletions, and the recessive allele was segregating at a low allele frequency (MAF for *FANCI* deletion = 0.06, MAF for *TFB1M* deletion = 0.005). Additionally, we detected a singleton pLoF 50-Kb deletion affecting *CENPC*, shown to result in an early embryonic loss in knock-out mice [25]. As such, mapping deleterious variants using statistical association may not be suitable for the current data set, yet exploiting a wealth of annotation data in human and mouse can shed light into functional interpretation of the gene-disrupting SVs reported in this study.

We mapped two copy gain events, overlapping with *ORM1* and *POPDC3*, respectively, to be promising SV-eQTL (Fig. 5 and Figure S10). *ORM1* encodes acute phase protein and is involved in energy metabolism: mice lacking *ORM1* expression were shown to have increased body weight and fat mass [26], whereas upregulation of *ORM1* in postpartum cows was correlated with reduced feed intake [27, 28]. Based on these studies, we had expected to find feed intake QTL coinciding with the *ORM1* duplication, however, no QTL was reported in the Animal QTL database or a GWAS study [42, 43]. There are several explanations for this conundrum. One possibility is that the duplication itself or tagging SNPs are inaccurately genotyped, leaving no association signals. Another possibility may have to do with the transient expression of *ORM1*. *ORM1* is strongly upregulated from parturition to postpartum day 14, hence suppressing feed intake during this short period. However, in breeding programmes, feed intake traits are defined as an overall mean during the lactation [44]. Thus, the suppressed feed intake during the first ~ 2 weeks may be diluted in such trait definition. As such, to measure the phenotypic impact of the *ORM1* duplication, a novel feed intake trait, limited to feed intake during ~ 2 weeks postpartum, may be highly relevant. Additionally, it is remarkable that the *ORM1* duplication is segregating at a high frequency despite its presumably negative impact (low postpartum feed intake), hinting that it might be under balancing selection. As with the feed intake QTL, we have not found any QTL associated with other traits, which may be logical if association studies did not have tagging SNPs capturing this duplication. Interestingly, *ORM1* duplication was reported in human populations as well. Diploid CNs of *ORM1* is highly frequent in the European population (CN > 10) compared to the African population (CNs 2–3) [7], suggesting that upregulation of *ORM1* might confer a generic adaptive advantage across species.

## Conclusion

This study reports a high-quality SV catalogue containing 13,925 SVs detected in a whole genome sequenced dairy cattle family cohort. Using the direct genotyping approach, we genotyped a subset of CNVs in an independent cohort and confirmed that 62% of the targeted CNVs are segregating in the population. In search of high impact SVs, we prioritised two duplications overlapping with *ORM1* and *POPDC3*, associated with feed intake and hoof health traits, respectively. The eQTL mapping results corroborate that these duplications are likely the causal variant driving the gene expression, underpinning the functional importance of SVs. Given the functional impact of SVs, incorporating them in large scale genetic analyses would be crucial. Yet, our LD analyses showed that most CNVs are not captured by 50K SNPs, stressing the importance of incorporating CNVs into routine analyses either by directly genotyping or exploiting CNV tagging SNPs. Also, a future study may investigate whether a haplotype-based approach outperformed tagging SNP approach. The current high-quality SV catalogue will serve as an invaluable resource for future population genetics studies.

## Methods

### Whole genome sequencing data

The genomes of 266 Dutch HF animals were sequenced. These 266 animals were closely related animals, where 240 were forming 127 parents-offspring trios. The biological materials were either from sperm (males) or whole blood (females and males). Whole genome Illumina Nextera PCR free libraries were constructed (550-bp insert size) following the protocols provided by the manufacturer. Illumina HiSeq 2000 instrument was used for sequencing, with a paired-end protocol (2x100bp) by the GIGA Genomics platform (University of Liège). The data was aligned using BWA mem (version 0.7.5a) [45] to the bovine reference genome ARS-UCD1.2 [46], and converted into bam files using SAMtools 1.9 [47]. Subsequently, the bam files were sorted with Sambamba (version 0.6.6) [48] and PCR duplicates were removed with Picard (version 2.7.1). All samples had a minimum mean sequencing coverage of 15X, and the mean coverage of the bam files was 26X.

### Structural variation discovery pipeline

We discovered SVs using Smoove pipeline (https://github.com/brentp/smoove). This pipeline collects split and discordant read pairs using Samblaster [49] and then discovers SVs per sample (step 1). The SV discovery was done sample by sample, using Lumpy [15], which detects deletions, duplications, inversions, and breakends (non-canonical forms of SVs; step 2). The per sample SV discovery showed that the number of SVs discovered per sample was related to the sequencing coverage (Figure S1). We did not find any outlier samples in terms of the total number of SV per sample and the number of singleton SVs per sample. Hence, the entire cohort of 266 animals was kept for further analysis. After the sample level SV discovery, all SVs were merged, creating a population-wide non-redundant SV call set (step 3). Subsequently, the entire cohort was genotyped for the non-redundant SV sites using SVTyper (https://github.com/hall-lab/svtyper), thus generating one vcf file per sample (step 4). Additionally, the step 4 included the annotation of the fold-coverage change of read-depth in SV using Duphold [16]. Duphold annotated two read-depth values: (i) DHFFC representing sequencing depth fold-change for the variant compared to 1-kb flanking regions, and (ii) DHBFC representing sequencing depth fold-change for the variant compared to genomic regions with similar GC-content. We used DHFFC for filtering deletions and DHBFC for filtering duplications, as recommended by the developer. Finally, the individual vcf files were merged into a multi-sample vcf file (step 5). This multi-sample vcf file contained non-redundant 38,094 SVs (17,826 deletions, 4,652 duplications, 1,811 inversions, 13,805 breakends).

### Establishing a clean call set exploiting the post hoc filtering based on read-depth changes

The initial SV call set contained 38,094 non-redundant SVs (17,826 deletions, 4,652 duplications, 1,811 inversions, 13,805 breakends). We filtered out the following:

(i) 1,811 inversions and 13,805 breakends; retaining 17,826 deletions and 4,652 duplications.
(ii) 495 deletions smaller than 50-bp; retaining 17,331 deletions and 4,652 duplications.
(iii) 193 deletions and 11 duplications that were not polymorphic in the current population (all animals genotyped as 0/0 or 1/1); retaining 17,138 deletions and 4,641 duplications.
(iv) 42 deletions suggesting assembly issues (all animals genotyped as 0/1); retaining 17,096 deletions and 4,641 duplications.
(v) 4,405 deletions and 3,037 duplications suggesting false calls based on spurious read-depth fold-coverage changes (explained below); retaining 12,691 deletions and 1,604 duplications.

Current SV call set was genotyped based on SR and DP evidence (reads-based genotyping). In contrast, CNVs can be genotyped solely relying on changes in read-depth in absence of SR and DP evidence. Thus, to refine our SV call set, we performed a post hoc site-level filtering using the read-depth fold-coverage annotation obtained from Duphold (step 4 of the pipeline; see above). We obtained the mean read-depth fold-coverage values per genotype and SV type. The mean read-depth fold-coverage values for deletions were 0.91 for 0/0; 0.55 for 0/1 and 0.03 for 1/1. For duplications, 1 for 0/0, 1.22 for 0/1, and 1.45 for 1/1. Subsequently, we generated a QC plot per SV which visualizes the read-depth fold-coverage change depending on genotypes (Fig. 1D). By manually inspecting underlying WGS data and the QC plots of all SVs on chromosome 1, we established rules that for deletions, a site should meet the following: (a) the ratio between mean read-depth fold-coverage in 0/0 carriers and mean read-depth fold-coverage in 0/1 carriers should be larger than 1.39, and (b) neither mean read-depth fold-coverage in 0/0 carriers nor mean read-depth fold-coverage in 0/1 carriers is equal to 0, and (c) the highest read-depth fold-coverage in 0/0 carriers is smaller than 3, and (d) the highest read-depth fold-coverage in 0/1 carriers is smaller than 1.3, and (e) the lowest read-depth fold-coverage of 0/0 carriers are larger than 0.1; for duplications, a site should meet the following: (a) the ratio between mean read-depth fold-coverage of 0/1 and1/1 carriers and read-depth fold-coverage of 0/0 carriers should be higher than 1.2 and smaller than 5, and (b) the lowest read-depth fold-coverage in 0/0 carriers should be higher than 0.44, and (c) the lowest read-depth fold-coverage in 0/1 carriers should be higher than 0.5.

(vi) 490 deletions and 74 duplications that were duplicates (explained below); retaining 12,201 deletions and 1,530 duplications.

While inspecting all SVs on chromosome 1, we discovered that our pipeline sometimes outputs two call (e.g., due to complex breakpoints) although there is a single true SV. We manually screened all SVs using IGV to detect duplicate SVs.

Finally, some duplication QC plots showed non-canonical read-depth fold-coverage changes. For a biallelic duplication, read-depth fold-coverage changes will form three distinctive distributions (Fig. 1D). Some duplications showed non-canonical form of read-depth fold-coverage distributions, which formed more than three distributions, indicating more than two alleles. By manually screening all QC plots, we re-classified 21 duplications as mCNVs (Figure S2). With this our clean call set was established, which includes 12,201 deletions, 1,509 duplications, and 21 mCNVs.

### Differentiating the clean call set into stringent and lenient calls

While manually inspecting underlying WGS data and the QC plots of all SVs on chromosome 1, we learned that some clean SV calls do not conform canonical read-depth fold-coverage changes. For example, for a deletion, one may be genotyped as 0/0, despite the fact that the read-depth fold-coverage indicates heterozygous deletion (e.g., 0.5). For downstream analyses which may require accurate genotyping results, we divided the clean SV call set into a “stringent” call set, with CNVs of which their read-depth fold-coverage corresponds unambiguously to the reads-based genotypes, and a “lenient” call set with CNVs of which their read-depth fold-coverage does not always match with the reads-based genotypes. Among the CNVs in the clean call set, ones that meet the following criteria were classified as stringent and otherwise as lenient.

(i) For deletions, (a) the highest read-depth fold-coverage value among 0/1 carriers should be smaller than the lowest read-depth fold-coverage value among 0/0 carriers, and (b) the highest read-depth fold-coverage value among 0/0 carriers should be smaller than the lowest read-depth fold-coverage value among 0/1 carriers, and (c) the lowest read-depth fold-coverage value among 0/1 carriers should not be equal to 0;
(ii) For duplications, (a) the lowest read-depth fold-coverage value among 0/1 carriers should be larger than the highest read-depth fold-coverage value among 0/0 carriers, and (b) the lowest read-depth fold-coverage value among 1/1 carriers should be larger than the highest read-depth fold-coverage value among 0/1 carriers, and (c) the lowest read-depth fold-coverage value among 0/1 carriers should not be equal to 0, and (d) the lowest read-depth fold-coverage value among 1/1 carriers should not be equal to 0;

Using these criteria, we classified the clean call set into 4,013 stringent calls (3,828 deletions and 185 duplications) and 9,697 lenient calls (8,373 deletions and 1,324 duplications).

### Evaluation of the SV call set

#### Mendelian inheritance errors

Using the 127 trios, we coined a quality assessment metric based on Mendelian inheritance. We counted the number of trios showing Mendelian inheritance error and expressed it as a fraction. For example, for a CNV locus, if 10 trios showed Mendelian error, we assigned 0.08 (10/127 = 0.08). Hence, the scale ranged from 0 to 1, where 0 stands for no trios showing inheritance error, whereas 1 indicates all of the 127 trios showing inheritance error. We calculated this metric for all the CNV sites, both lenient and stringent calls. Additionally, to correct for a possible bias, where common CNVs may be biased to have higher Mendelian errors, we changed the denominator from the total number of trios (n = 127) to the effective number of trios of which at least one of the trio animals carried the variant for a given site.

#### Direct genotyping of CNVs using a 50 K SNP array

To avoid highly costly validations, we opted for direct genotyping of a subset of CNVs. We designed probes directly targeting the breakpoint sequences of 372 CNVs (342 deletions and 30 duplications) that appeared in non-repetitive regions, using the Illunima DesignStudio Custom Assay Design Tool. These probes were added in the custom part of the EuroGenomics SNP genotyping array [18]. Genotyping was done for 815 Dutch HF animals using their ear punch or blood samples. Of note, these samples did not overlap with the WGS data set samples.

#### Comparison with an array-based CNV catalogue

The overall WGS-based CNV call set (including both lenient and stringent calls) was compared to an array-based CNV call set. The array-based CNV call set was obtained based on Illumina BovineHD Genotyping BeadChip (770 K) in 315 HF animals [14]. Of the 315 animals, 34 were overlapping with the WGS samples. The event size determined from an array-based CNV detection is strongly dependent on the local probe density. Thus, applying reciprocal overlap criteria for comparing array- and WGS- call set may underestimate the true overlapping calls. Accordingly, we intersected two call sets using Integrative Genome Viewer (IGV) [50] and manually inspected the underlying WGS data for overlapping calls. Where array- and WGS- based sites are overlapping and the underlying WGS data supports true presence of CNVs, we confirmed them as overlapping calls.

### Linkage disequilibrium in WGS data sets

We investigated the CNV-SNP LD using WGS data sets. SNPs were discovered from the same WGS data set explained above. Variant calling was done using GATK workflow (v4.1.7) and subsequently recalibrated using the following algorithms: BaseRecalibrator, HaplotypeCaller, GenomicsDBImport, GenotypeGVCF, GatherVcfs, Variant Recalibrator [51–53]. We applied Variant Quality Score Recalibration (VQSR) at a truth sensitivity filter level of 97.5 to remove spurious variants. For calculating CNV-SNP pairwise LD, SNPs located inside the CNVs were removed, and SNPs located within 100-kb distance from the CNV breakpoints were kept. Pairwise CNV-SNP LD (r^2^) was obtained from PLINK software (v1.9) [54].

### Linkage disequilibrium in 50 K SNP array data sets

The genotype data obtained from 50K SNP array, augmented with probes targeting the CNV breakpoints, was used to obtain 50K level CNV-SNP LD (explained above). Genotyping was done for 815 Dutch HF animals, and all samples passed the quality criteria (call rate per sample > 0.99). Of 53,917 SNPs and 284 CNVs that passed variant level filter (call rate per variant > 0.99), 50,342 SNPs and 229 CNVs were segregating in the population. As the number of segregating duplications was low (n = 19), we only performed the LD analyses on 210 deletions. We compared SNP-SNP and CNV-SNP LD depending on the inter-marker distance. The number of CNVs was lower than SNPs, and hence SNP-SNP pairs outnumbered CNV-SNP pairs. To compare the same number of pairs, we sampled an equal number of SNP-SNP pairs 1,000 times and compared the mean LD with the CNV-SNP pairs. Pairwise SNP-SNP and CNV-SNP LD (r^2^) was obtained from PLINK software (v1.9) [54]. The analyses were ran for common (MAF ≥ 0.05) and rare (MAF < 0.05) variants separately.

### Coding sequence disrupting SVs

We classified coding sequence (CDS) disrupting SVs into predicted loss-of-function, copy gain, and intergenic exon duplication following [22]. The CDS disrupting SVs were identified using Variant Effect Predictor (Ensembl release 98) [55].

### Regulatory element disrupting SVs

Bovine liver ChIP-seq data (H3K27ac and H3K4me3) was obtained from ArrayExpress (E-MTAB-2633; [31]). This ChIP-seq data was aligned to the bovine reference genome ARS-UCD1.2 using Bowtie2 [56], and peaks were called using MACS2 [57]. The SVs overlapping with enhancer or promoter signals were identified using BedTools software [58]. Based on the strength of the regulatory elements signal, the allele frequency of the SVs (MAF > 0.05), and literature suggesting their functional roles in phenotypes [27, 28, 30], we selected two SVs for subsequent SV-eQTL mapping.

### SV-eQTL mapping

#### Genotype data and imputation

Liver biopsy samples were collected from ~ 14-day postpartum HF cows (n = 178). The procedures had local ethical approval and complied with the relevant national and EU legislation under the European Union Regulations 2012 (S.I. No. 543 of 2012). These samples were genotyped using Illumina BovineHD Genotyping BeadChip (770K). Genotypes in a 10-Mb region encompassing the SV of interest were parsed out, and rare variants were filtered out (MAF < 0.02). We included both SNPs and the SVs of interest in the imputation panel to see whether SV(s) is the underlying variant driving the gene expression. The SNPs were discovered, as explained above. Two different SV genotypes were used: (i) the original reads-based genotypes obtained from SVtyper (https://github.com/hall-lab/svtyper) which utilizes SR and DP evidence and (ii) genotypes manually corrected based on the read-depth fold-coverage changes (Figure S11). The initial imputation panel included SNPs in a 10-Mb region harbouring the SV of interest and two different genotypes of SVs (contained 266 WGS animals). This panel was phased, and variants with low phasing accuracy and allele frequency were filtered out (DR2 < 0.95 and MAF < 0.02). Subsequently, the BovineHD genotypes were imputed to the sequence level variants and variants with low imputation accuracy and low minor allele frequency were filtered out (allele R^2^ < 0.9 and MAF < 0.025). Phasing and imputation were done using Beagle 4 [59].

#### RNA-seq data and eQTL mapping

The liver RNA-seq data was obtained from the GplusE consortium (http://www.gpluse.eu; EBI ArrayExpress: E-MTAB-9348 and 9871) [60]. RNA-seq libraries were constructed using Illumina TruSeq Stranded Total RNA Library Prep Ribo-Zero Gold kit (Illumina, San Diego, CA) and sequenced on Illumina NextSeq 500 sequencer with 75-nucleotide single-end reads to reach average 32 million reads per sample. The reads were aligned to the bovine reference genome ARS-UCD1.2, and its corresponding gene coordinates from UCSC as a reference using HISAT2 [61]. Transcript assembly was conducted with StringTie [62], using a reference-guided option for transcript assembly. Reads were counted at gene level using StringTie. Subsequent QC on the RNA-seq data set removed three samples with suboptimal quality (QC steps are explained in detail elsewhere; [17]), normalised gene expression was associated with the imputed WGS variants for 175 samples, using a linear model in R package “MatrixEQTL” [63].

## Electronic supplementary material

Below is the link to the electronic supplementary material.

**Additional file 1: Figure S1.** Number of discovered SVs depending on mean sequencing depth. **Figure S2.** Distinctive RD distribution discerning mCNVs from biallelic duplications. **Figure S3.** Size distribution of overall CNVs. **Figure S4.** Canonical and non-canonical copy number variants. **Figure S5.** Number of variants before and after filtering steps. **Figure S6.** Mendelian error fraction obtained from the effective number of trios. **Figure S7.** Minor allele frequency between the discovery and validation cohort. **Figure S8.** Maximum LD (r2) between CNV-SNP pairs in WGS data set. **Figure S9.** Underlying WGS data for a 16-kb mCNV inspected in IGV. **Figure S10.** Underlying WGS data for two copy gain duplication events. **Figure S11.** Manual correction of ORM1 duplication genotypes.

**Additional file 2: Supplementary table 1.** A list of structural variants identified in the current study and their cooridnates, length, type, category, allele frequency, whether a SV was validated or not via the 50K SNP genotyping approach, and genic overlap are shown in the table. **Supplementary table 2.** A list of 127 trios and the corresponding animal IDs for each trio.

## Data Availability

All sequence data of the 266 Dutch Holstein Friesian animals are deposited in the European Nucleotide Archive under accession PRJEB53518/ERA15565221. The RNA-seq data is deposited under EBI ArrayExpress accession E-MTAB-9348 and 9871.
