# Supplementary material for: High-resolution structural variants catalogue in a large-scale whole genome sequenced bovine family cohort data
Source: BMC Genomics. 2023 May 1;24:225. doi: 10.1186/s12864-023-09259-8 (PMC10152703; doi:10.1186/s12864-023-09259-8)
Supplement: Supplementary file 1 — Additional file 1: Figure S1. Number of discovered SVs depending on mean sequencing depth. Figure S2. Distinctive RD distribution discerning mCNVs from biallelic duplications. Figure S3. Size distribution of overall CNVs. Figure S4. Canonical and non-canonical copy number variants. Figure S5. Number of variants before and after filtering steps. Figure S6. Mendelian error fraction obtained from the effective number of trios. Figure S7. Minor allele frequency between the discovery and validation cohort. Figure S8. Maximum LD (r2) between CNV-SNP pairs in WGS data set. Figure S9. Underlying WGS data for a 16-kb mCNV inspected in IGV. Figure S10. Underlying WGS data for two copy gain duplication events. Figure S11. Manual correction of ORM1 duplication genotypes. [file 12864_2023_9259_MOESM1_ESM.docx]

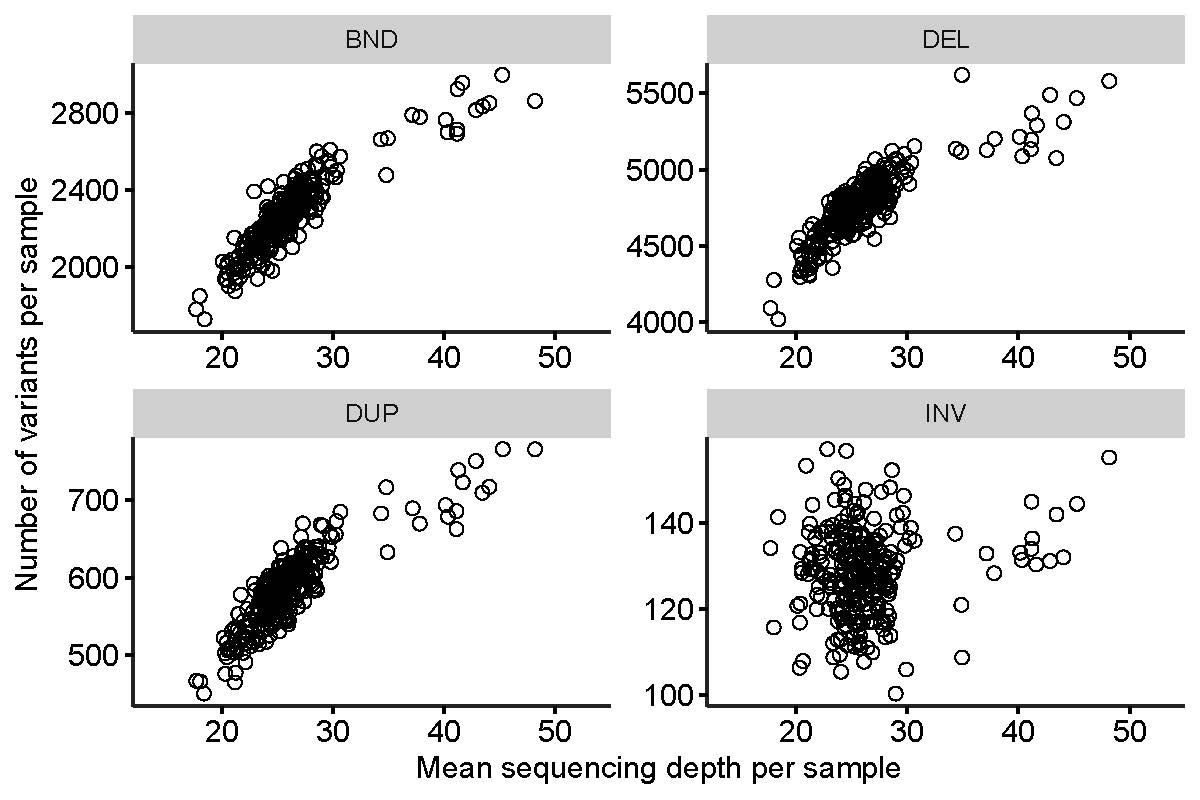


**Figure S1.** **Number of discovered SVs depending on mean sequencing depth**

Our SV discovery results showed that the number of SVs discovered per sample increased linearly in relation to the mean sequencing depth. Each panel stands for four types of SVs: BND (breakends), DEL (deletions), DUP (duplications), and INV (inversions). Each dot represents a sample. For BND, DEL, and DUP, the increase of discovered variants according to the sequencing depth was evident. We did not observe spurious batch effects. There were no spurious sub-clusters (e.g. batch effects).


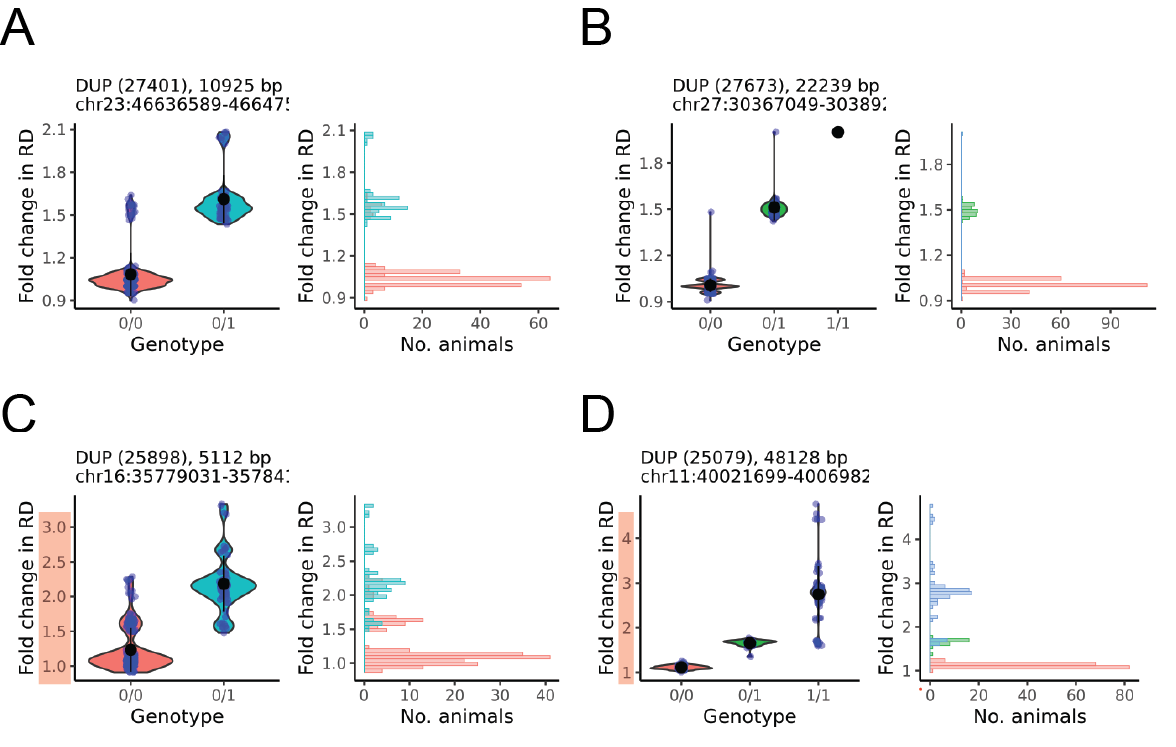


**Figure S2.** **Distinctive RD distribution discerning mCNVs from biallelic duplications**

We re-classified 22 duplications as mCNVs based on their RD distribution. **(A, B)** Panel A and B show examples of biallelic duplication sites where RD values form the trimodal distribution. **(C, D)** In the case of multi-allelic duplications, there are more than three allelic combinations in diploid states and hence show distinctive multi-modal RD distribution. Also, the range of RD values is broader than biallelic duplications (x-axes marked with translucent red).


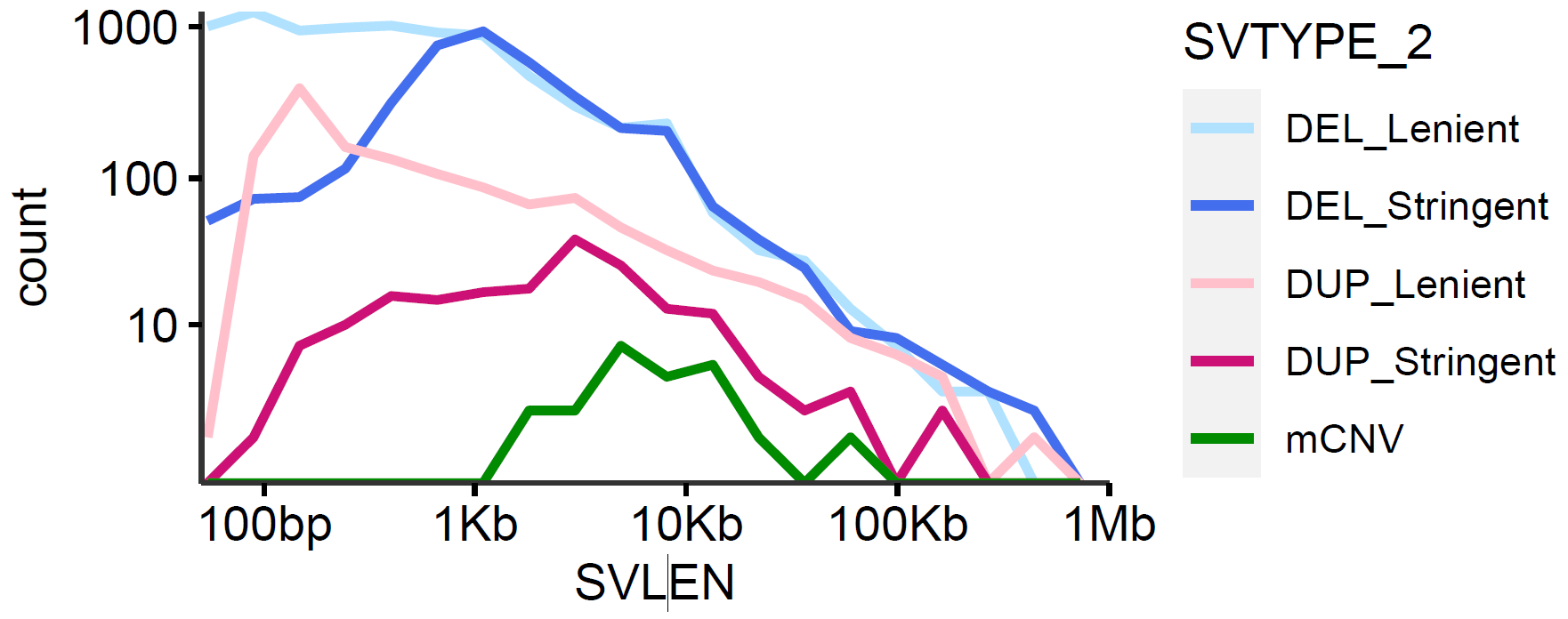


**Figure S3. Size distribution of overall CNVs**

Size distribution of overall CNVs are shown (deletions, duplications, and mCNVs). The deletions are marked with light blue (lenient calls) and dark blue (stringent calls). The duplications are marked with light pinks (lenient) and magenta (stringent). mCNVs are marked with green and they are discovered in >1-kb size.


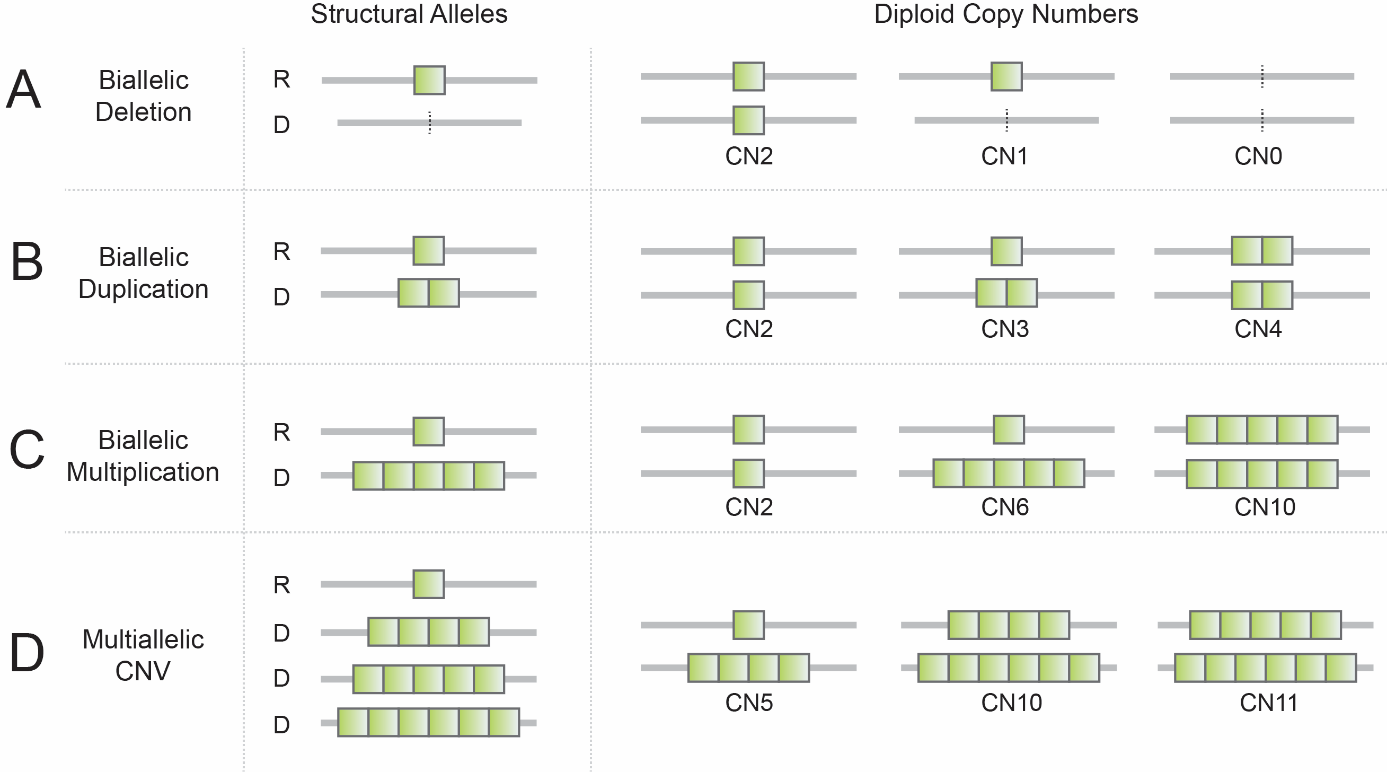


**Figure S4. Canonical and non-canonical copy number variants.**

The figure above provides a schematic overview of the canonical and non-canonical CNVs. The left side shows alleles segregating at a given locus and the reference allele is marked with (R) and the derived allele is marked with (D). The right panel shows the allelic combination possible for a given locus, and the diploid copy numbers. Canonical CNVs include biallelic deletions and duplications which harbours two structural alleles for a given locus (for deletions wildtype and deleted; for duplications wildtype and duplicated). Multiplications refers to SVs of which the derived allele does not show sequential copy number increase. Hence, unlike the biallelic duplication which will have diploid CN 2, 3, and 4 (B), multiplication loci can have higher diploid CNs (C). We learned that the genotyping tool used in the current study assumes two alleles for any SVs by default. Hence, often multiplication loci, classified as duplications, show high degree of Mendelian errors. Furthermore, some CNVs harbour more than two structural alleles, thus enabling many more allelic combinations and diploid CNs (D).
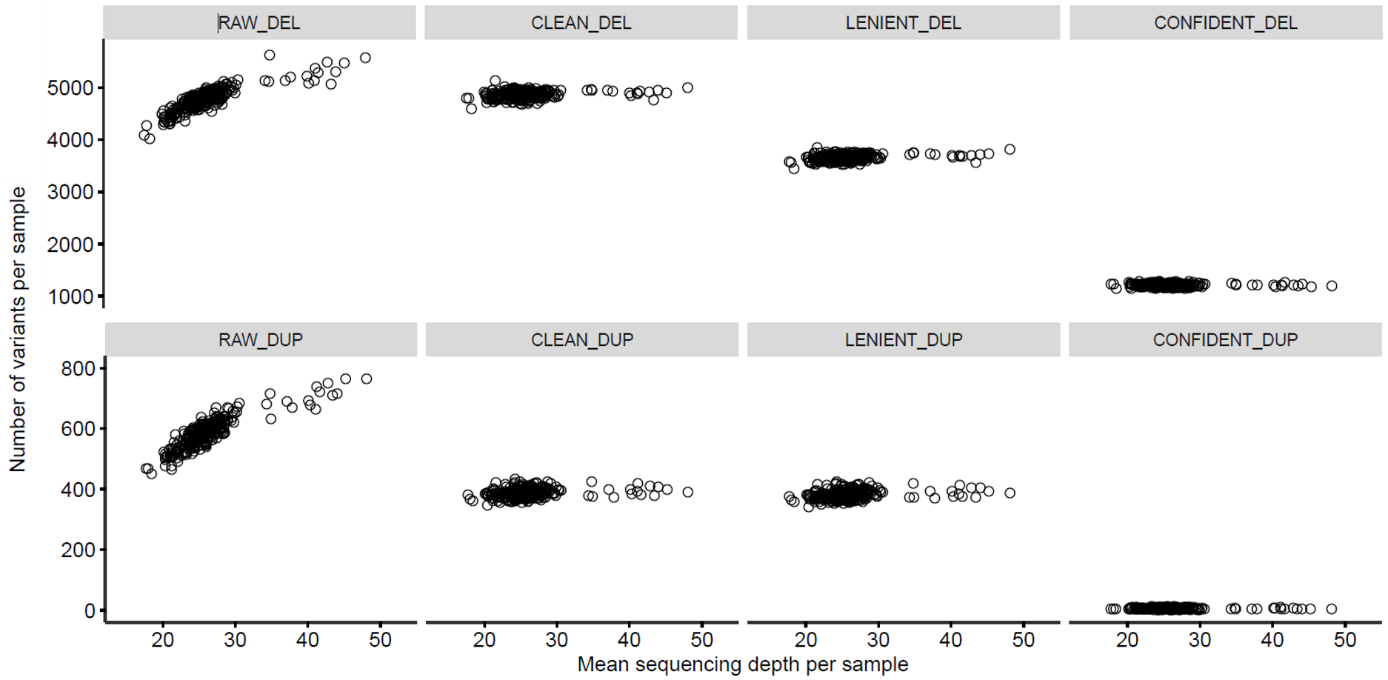


**Figure S5. Number of variants before and after filtering steps**

The number of variants (deletions and duplications) discovered per sample, displayed in a function of sequencing depth. In the initial discovery phase, the number of discovered variants increase along with the sequencing depth. After the filtering steps (see materials and methods) the clean call set was established. For the clean call set, the number of variants per sample is not strongly affected by the sequencing depth of the sample. This indicates that a large fraction of increase in the detected variants in the raw call set was false SV calls. Afterwards, the clean call set was divided into stringent and lenient call sets. These two call sets also did not display dependence on the sequencing coverage, underlining that the filtering steps effectively removed false calls that were prone to high sequencing coverage samples.


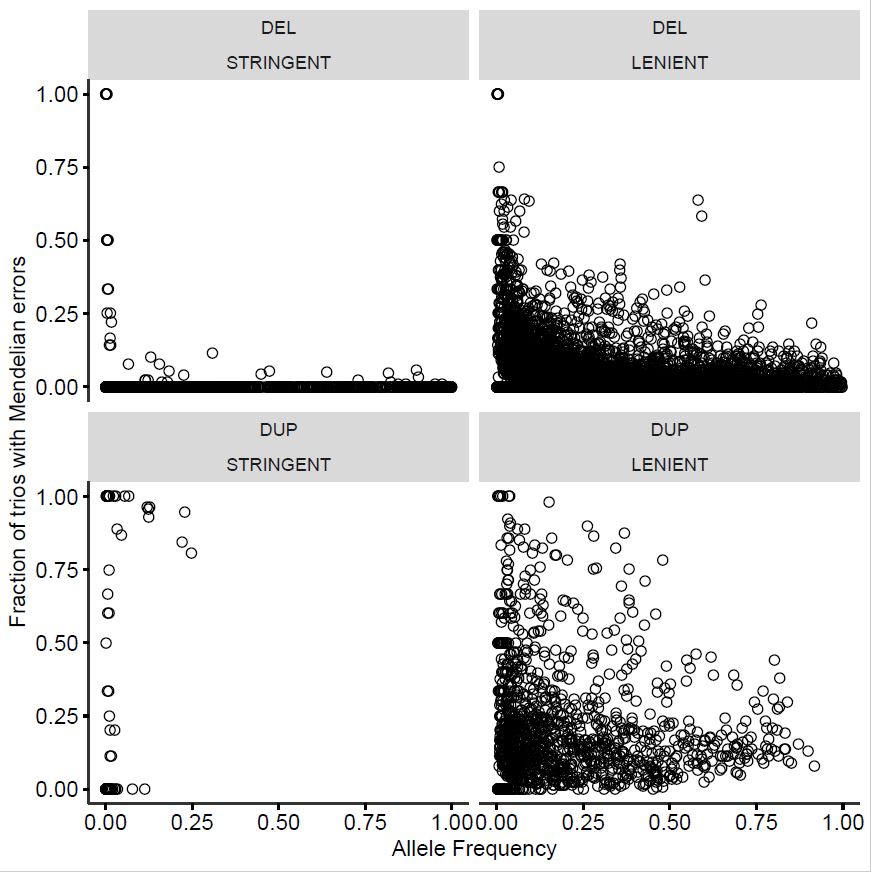


**Figure S6. Mendelian error fraction obtained from the effective number of trios**

The Mendelian error fraction was initially obtained by dividing the number of trios expressing Mendelian errors by the total number of trios (n=127). As this approach can deflate the error fraction in rare variants, whereas inflate the error fraction in common variants. To correct for this potential bias, we divided the number of trios showing Mendelian errors, by the number of trios of which at least one animal is variant for the site of interest. Hence, if none of the trio animals is variant (i.e. sire, dam, and proband are 0/0 for a site of interest), we did not count. Using this approach which only takes into account the effective number of trios, we did not see that the Mendelian error fraction increases along with the allele frequency for deletions. When it comes to duplications, they were more variable and showed generaly higher Mendelian error fraction, which was expected due to (i) multiplications, (ii) mCNVs, and (iii) generally low genotyping accuracy compared to deletions.


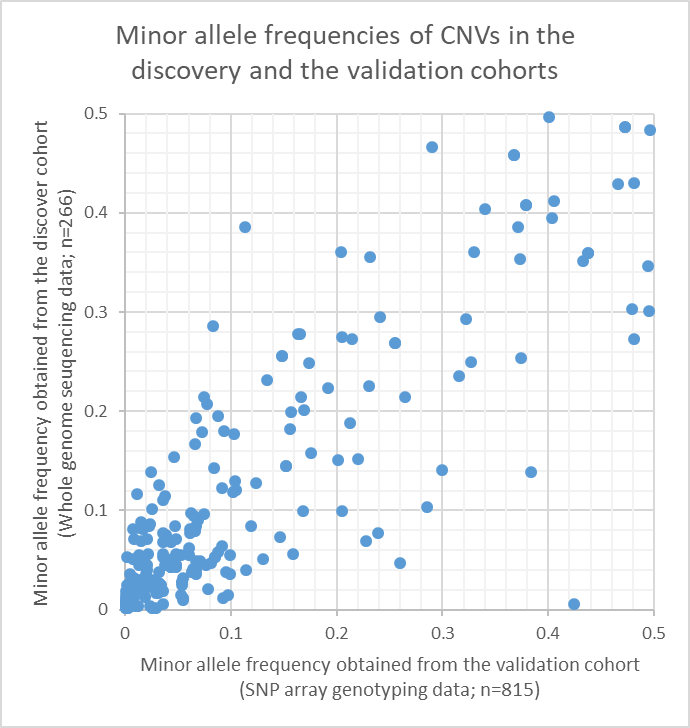


**Figure S7. Minor allele frequency between the discovery and validation cohort**

We compared the minor allele frequencies of the CNVs validated via direct genotyping approach. Except few minor outliers, most CNVs showed similar minor allele frequencies in the discovery and the validation cohorts.


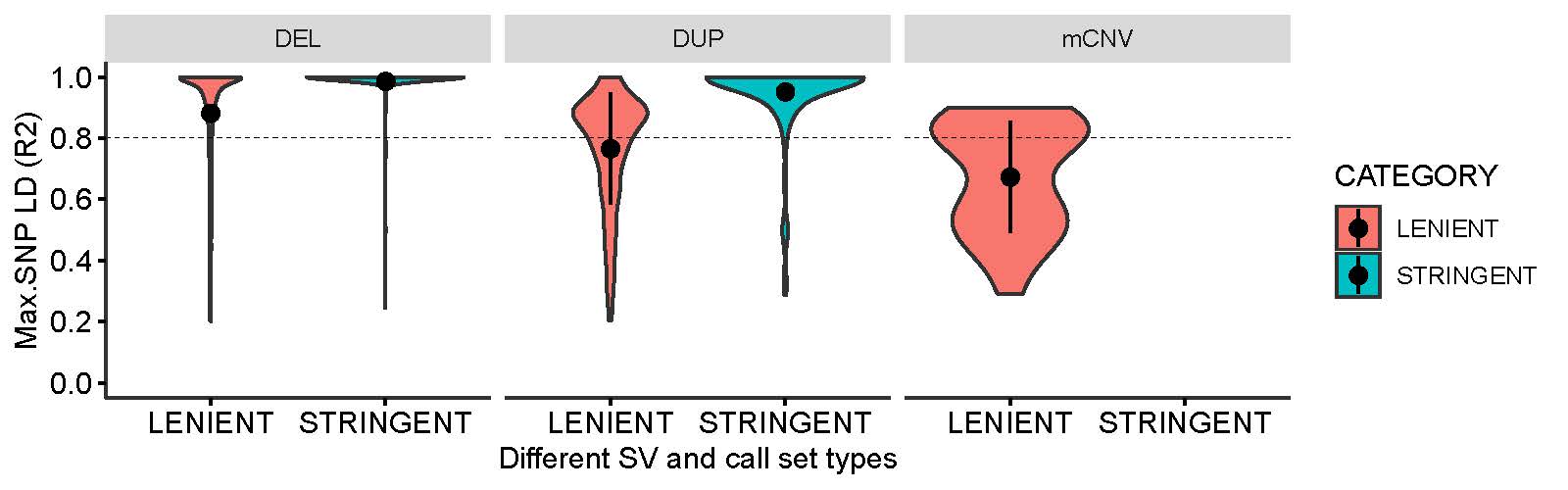


**Figure S8. Maximum LD (r2) between CNV-SNP pairs in WGS data set**

We took the maximum LD (r2) a CNV has between SNPs located within 100-Kb distance. For both deletions and duplications, stringent calls have a higher maximum R2 than those in the lenient call set. mCNV showed overall lower LD than biallelic CNVs.


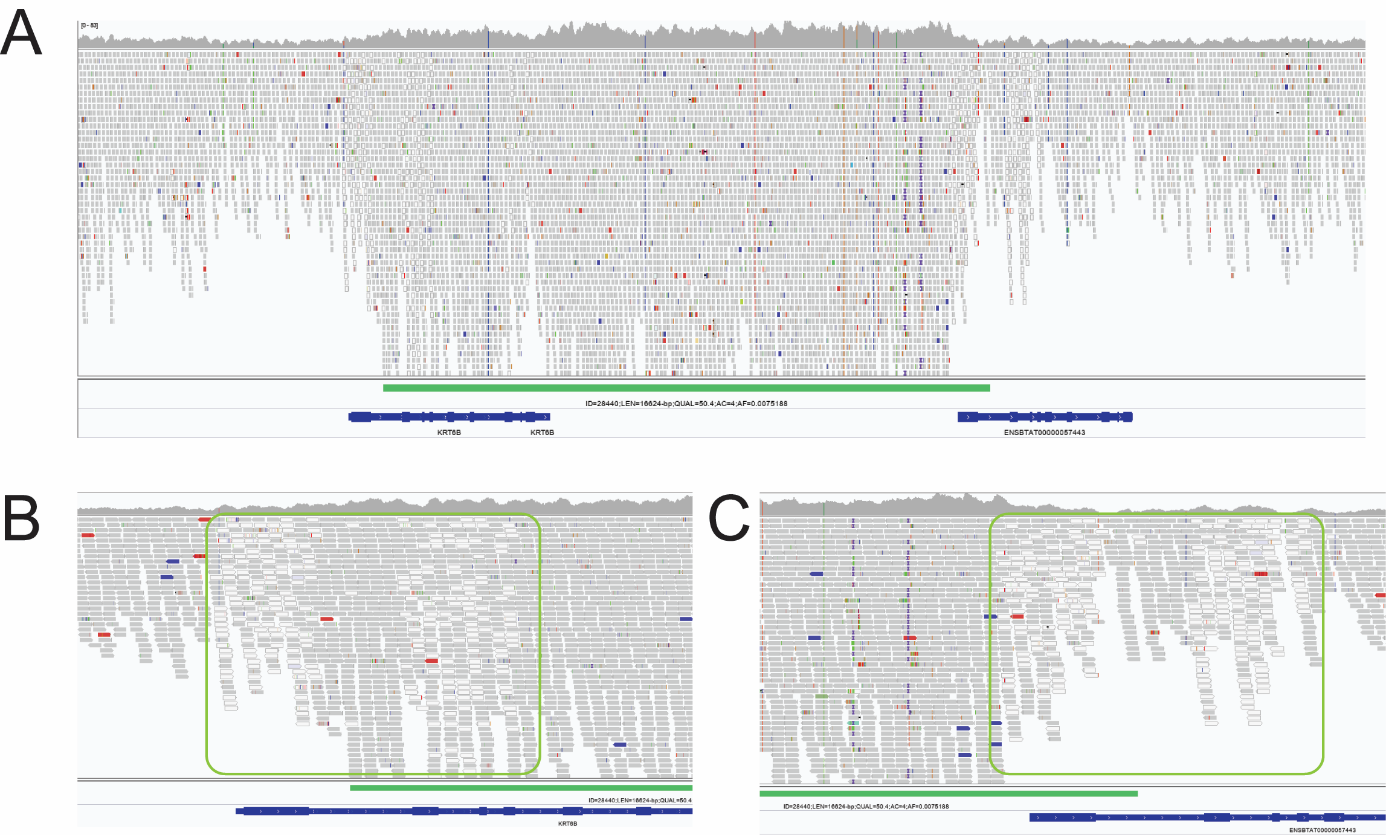


**Figure S9. Underlying WGS data for a 16-kb mCNV inspected in IGV**

**(A)** The underlying WGS data of the 16-kb mCNV was inspected. The green bar indicates the mCNV, and the lower bar shows two keratin genes disrupted by the mCNV. **(B)** The proximal breakpoint of the mCNV. The grey colored bars indicate reads with high mapping quality. The white bars in the green box indicate reads with low mapping quality. **(C)** The distal breakpoint of the mCNV. The legends are identical to panel C.


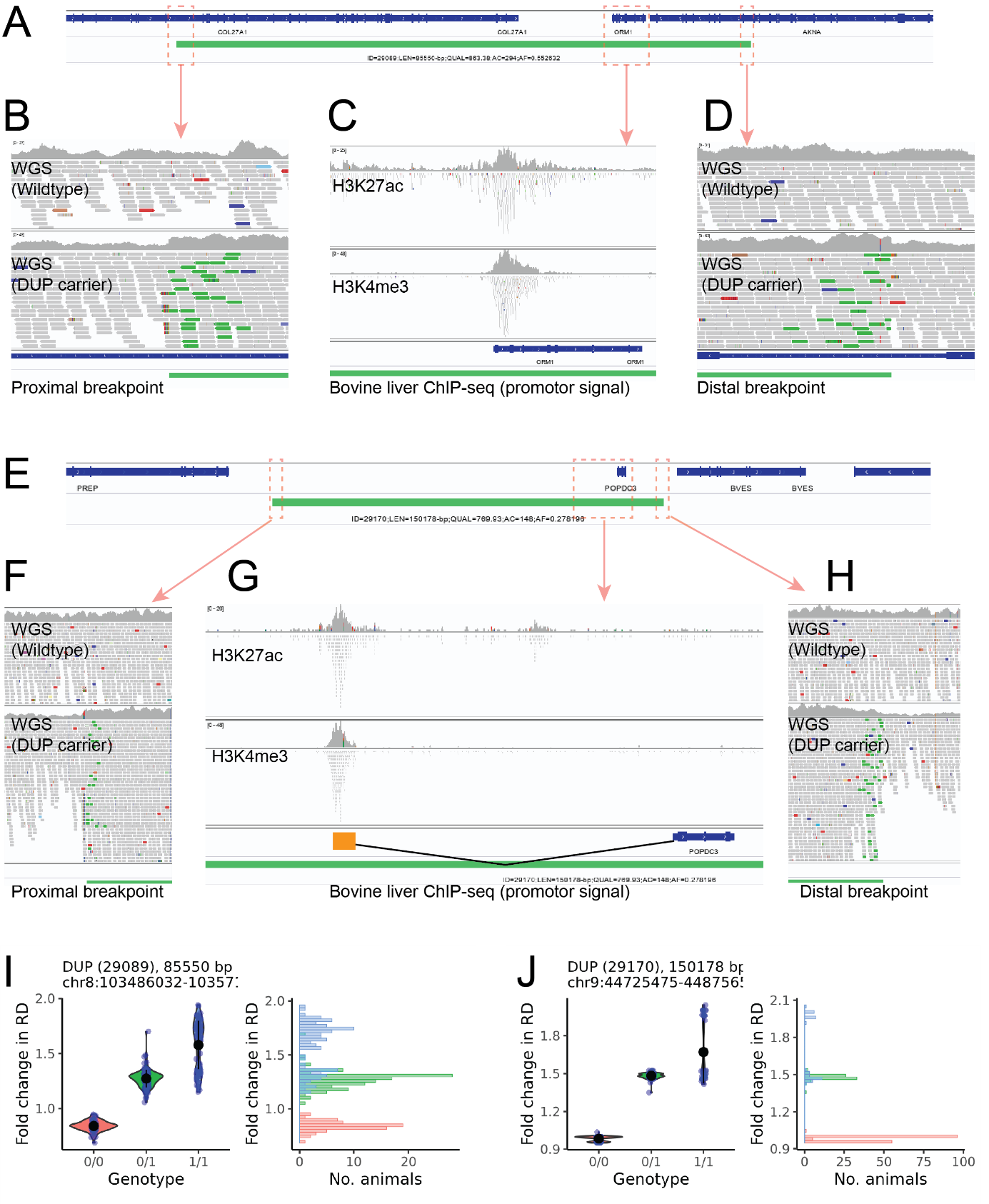


**Figure S10. Underlying WGS data for two copy gain duplication events**

**(A)** A schematic overview of the *ORM1* duplication (marked with green) and the overlapping genes (gene structure shown in dark blue). **(B)** The proximal breakpoint of the *ORM1* duplication zoomed-in in IGV. Grey color reads stand for well-aligned ones, whereas green ones stand for discordant ones indicating tandem duplications. **(C)** The bovine liver ChIP-seq data (H3K27ac and H3k4me3) and the gene annotation were inspected. The ChIP-seq signals shown at the start of the *ORM1* supports the presence of a liver promoter. **(D)** The distal breakpoint of the *ORM1* duplication is zoomed-in IGV. The legends are identical to panel B. **(E)** A schematic overview on the *POPDC3* duplication (marked with green) and the overlapping gene (gene structure shown in dark blue). **(F)** The proximal breakpoint of the *POPDC3* duplication is zoomed-in IGV. The legends are identical to panel B. **(G)** The bovine liver ChIP-seq data (H3K27ac and H3k4me3) and the gene annotation were inspected. The ChIP-seq signals indicating a liver promoter appeared upstream of the *POPDC3*. We confirmed an unannotated exon of the *POPDC3* gene coinciding with the ChIP-seq signal (unpublished data). **(H)** The distal breakpoint of the *POPDC3* duplication is zoomed-in IGV. The legends are identical to the panel B. **(I,J)** QC plots for the *ORM1* duplication and *POPDC3* duplication, respectively. In both cases, the RD distribution of GT 0/1 and GT 1/1 are overlapping, indicating inaccurate genotyping results.


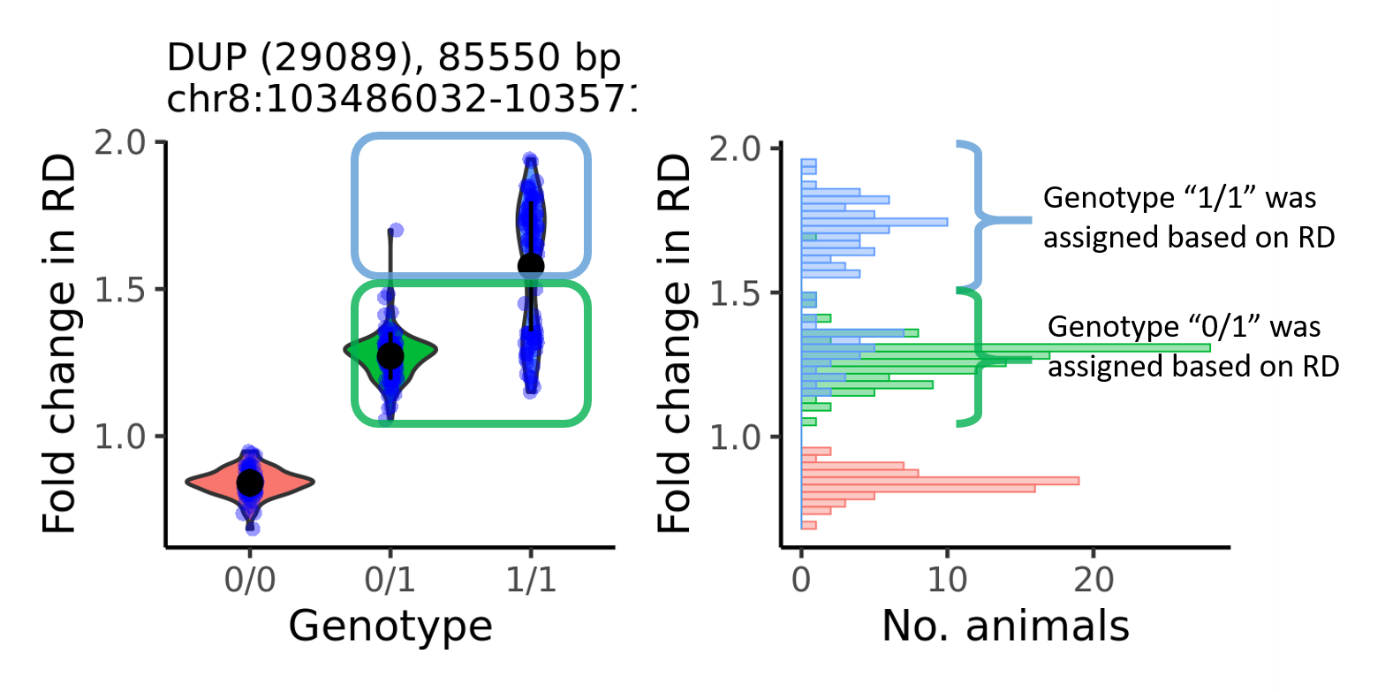


**Figure S11. Manual correction of ORM1 duplication genotypes**

The plot above shows the standard QC plot generated for the ORM1 duplication. The three genotypes assigned by the read-based genotyping approach is marked by red for 0/0, green for 0/1 and blue for 1/1. The 1/1 animals form two distinctive clusters (left plot) indicating in accurate genotyping. Thus, we manually re-assigned RD-based GT by assigning 1/1 to the ones in the blue translucent box (left plot) and 0/1 to the ones in the green translucent box (left plot). The imputation of ORM1 duplication passed the QC threshold with this RD-based manual genotyping. The read-based genotyped ORM1 duplication did not meet the imputation quality criteria, hence discarded.
